# Phase I/II Trial of Sorafenib in Combination with Vinorelbine as First-Line Chemotherapy for Metastatic Breast Cancer

**DOI:** 10.1371/journal.pone.0167906

**Published:** 2016-12-19

**Authors:** Cristiano Ferrario, Ivan Strepponi, Khashayar Esfahani, Helen Charamis, Adrian Langleben, Emanuela Scarpi, Oriana Nanni, Wilson H. Miller, Lawrence C. Panasci

**Affiliations:** 1 Department of Medical Oncology, McGill University, Montreal, Canada; 2 Department of Cell Therapeutics, Sede Secondaria Della Cell Therapeutics, Bresso, Italy; 3 Department of statistics, Istituto Scientifico Romagnolo per lo Studio e la Cura dei Tumori, Rome, Italy; Fondazione IRCCS Istituto Nazionale dei Tumori, ITALY

## Abstract

**Background:**

Preclinical models have reported a synergistic interaction between sorafenib and vinorelbine. We investigated the toxicity, efficacy, and pharmacokinetics interaction of this combination as first-line treatment for patients with metastatic breast cancer.

**Methods:**

Patients were HER2-negative and treated with vinorelbine 30 mg/m^2^ IV days 1,8 every 21 plus daily oral sorafenib. In the phase I portion (3+3 design) patients received sorafenib 200 mg BID (cohort 1) or 400 mg BID (cohort 2). In the phase II expansion, 21 more evaluable patients were planned to receive the maximum tolerated dose (MTD). Pharmacokinetic analysis was performed in 6 patients: blood concentrations were compared for each drug in the presence or absence of the other drug.

**Results:**

In cohort 1, one patient experienced a dose-limiting toxicity (DLT) (grade 3 pancreatitis), requiring the expansion of this cohort to 6 patients, without further documented DLTs. In cohort 2, one patient of six experienced a grade 4 DLT (asymptomatic rise in amylase not requiring drug discontinuation), establishing this dose level as the MTD (sorafenib 400 mg BID). After expansion at the MTD, a total of 27 patients (median age 57) were treated for a median of 8 cycles. One grade 5 febrile neutropenia occurred. With repeated cycles, 52% of patients required at least 1 dose reduction of either drug. One patient experienced a sustained grade 3 fatigue resulting in treatment discontinuation. The response rate was 30%. Median PFS was 5.7 months (95% CI 4.4–7.6), and clinical benefit (absence of disease progression at 6 months) was 48%. PK analysis showed a significant interaction between the two drugs, resulting in a higher Cmax of vinorelbine in the presence of sorafenib.

**Conclusion:**

The combination of sorafenib and vinorelbine at full doses is feasible but not devoid of toxicity, likely also due to a significant PK interaction.

**Trial Registration:**

ClinicalTrials.gov NCT00764972

## Introduction

Metastatic breast cancer is a virtually incurable disease, for which aggressive treatments are not justified in most cases. Rather, there is a need to develop new combinations that might maintain a good efficacy profile, without significantly compromising quality of life.

Vinorelbine is a semisynthetic vinca alkaloid that inhibits microtubule polymerization and has shown significant clinical activity as salvage therapy for metastatic breast cancer (MBC) [1]. Vinorelbine is often administered on days 1 and 8 of 21-day cycles, with no decrease in the administered dose intensity compared to more frequent schedules. The main dose limiting toxicity (DLT) is hematological, specifically neutropenia, and the most commonly reported toxicity is neurological, mainly occasional paresthesia, diminution of deep-tendon reflexes, and constipation.

Sorafenib was originally identified as a strong inhibitor of C- and B-RAF and reduces basal phosphorylation of the downstream MAPK pathway in a panel of breast cancer cell lines [2]. The Raf/MEK/ERK pathway is a strong inducer of proliferative and antiapoptotic genes involved in drug resistance and potentiation of tumor metastasis [3]. Further characterization of the compound also confirmed potent inhibition of a spectrum of receptor tyrosine kinases mainly involved in angiogenesis, including VEGFR2, Flt-3, PDGFR-beta, and c-kit [4]. Sorafenib is thus expected to exert a combined targeted antiproliferative effect on tumor cells together with antiangiogenesis properties.

Favourable cytotoxic effects were reported after treatment of a broad spectrum of human cancer cell lines and xenografts, with sorafenib and several chemotherapeutic agents, including vinorelbine, suggesting a synergistic interaction [2,5]. Two preliminary reports of sorafenib as single agent in MBC showed clinical activity, with manageable toxicity profile consisting of mostly grade 1 cutaneous, constitutional, and gastrointestinal toxicity [6,7]. Sorafenib has also been safely combined with multiple chemotherapy agents.

We investigated the clinical safety and activity of vinorelbine and sorafenib (VS), defining the recommended doses for this combination and its efficacy as first-line in MBC patients.

A parallel pharmacokinetic (PK) study was also run, to investigate a possible interaction, as the metabolism of both sorafenib and vinorelbine depends on hepatic CYP3A isoenzymes.

## Materials and Methods

### Study design

This is single center study, conducted with the approval of the Jewish General Hospital Research Ethics Committee in September 2016. Due to an administrative oversight, the first patient was recruited before the trial was officially registered. However, the authors confirm that all ongoing and related trials for this drug/intervention are registered. Written informed consent was obtained from all patients. The study planned first a phase 1b, to define the recommended doses of the VS combination. With a traditional 3+3 dose escalation in subsequent cohorts, we tested increasing doses of sorafenib (with a fixed starting dose of vinorelbine) up to the recommended dose for sorafenib monotherapy: 200 mg bid (cohort 1) and 400 mg bid (cohort 2). In case of dose-limiting toxicity (DLT) observed in cohort 1, the protocol allowed for testing of a cohort –1 (200 mg qd).

Three patients were enrolled starting at the first dose level and observed until completion of cycle 1: if any patient experienced a DLT, up to three additional patients were to be enrolled at the same dose level.

The recommended dose for the second part of the study was the maximum tolerated dose (MTD), defined as the highest dose at which no more than one patient out of six would experience a DLT or, in the absence of a toxic dose, the dose of 400 mg bid.

In the second part of the trial (phase II) the cohort of patients treated at the MTD was expanded up to a total of 27 patients treated with this schedule of VS. Primary endpoint was overall response rate. Secondary endpoints included clinical benefit rate, progression-free survival and safety analysis.

### Patient selection

Women at least 18 years of age with histologically proven metastatic breast cancer and a life expectancy over 6 months were eligible for the study. Inclusion criteria included: HER-2 negative disease as defined by local testing, measurable disease by RECIST 1.0 criteria; ECOG performance status of 0–1, adequate bone marrow reserve, as well as normal heart, kidney and liver functions (creatinine ≤1.5 UNL, bilirubin ≤1.5 UNL, AST/ALT ≤2 UNL and a baseline left ventricular ejection fraction (LVEF) of at least 50%). Patients were excluded if they received previous chemotherapy for metastatic disease, any previous therapy with vinorelbine or any anti-angiogenic therapy, any breast cancer treatment in 4 weeks preceding enrolment, known central nervous system metastases, previous history of ischemic cardiovascular disease or thromboembolic events, and other uncontrolled relevant medical conditions.

### Treatment and procedures

Patients received vinorelbine 30 mg/m^2^ on days 1,8 every 21 days and sorafenib daily (200 mg bid in cohort 1 and 400 mg in cohort 2). Radiological imaging was repeated every two cycles and cardiac monitoring (12-lead EKG plus echocardiogram or MUGA scan) was also scheduled every 12 weeks. In the absence of disease progression or unacceptable toxicity, patients received combination VS therapy for up to 8 cycles, or up to 4 cycles after achieving best response. Patients not progressing during the combination treatment continued to receive maintenance sorafenib monotherapy (400 mg bid). Dose adjustments and delays based on observed toxicities were detailed in the protocol.

### Statistical considerations

From a pooled analysis of published data on monotherapy vinorelbine as first-line treatment for metastatic breast cancer, an overall response rate of 43% was selected as historical control. For the sample size calculation of the phase II portion of the study, we hypothesized that if the VS combination could lead to a response rate of 63% (20% over historical control), developing a randomized trial would have been reasonable. With a power of 80% to detect such an increase and a significance level (alpha) of 0.10, the minimum sample size needed was 27 evaluable patients. Kaplan-Meier estimates were used to analyze time-to-event data.

For PK analysis, nonparametric Wilcoxon signe-rank test was used to compare the profile of each drug (and the M2 active metabolite of sorafenib) in the absence or presence of the other drug.

### Pharmacokinetics

Plasma samples for PK were collected in six patients treated at the recommended dose and analyzed by Bayer using validated liquid chromatography–mass spectrometry (LC-MS/MS) assays. These patients started taking sorafenib on day 4 of the first cycle, allowing us to compare the plasma levels of vinorelbine, sorafenib and M2 (N-oxide active metabolite of sorafenib) when vinorelbine and sorafenib were administered concomitantly and when they were given apart from each other.

Plasma samples for vinorelbine were collected on day 1 of the first cycle (before sorafenib treatment, starting on day 4) and on day 1 of cycle 2, in the presence of steady state levels of sorafenib. In both cases, collection time points for samples were: time 0 (pre-dose), at the end of the infusion, 0.5, 1, 2.5, 5, 7, 24, 48, 72 hours from the end of the infusion.

Samples for sorafenib and M2 were collected with a similar schedule (0, 0.5, 1, 2.5, 5, 7, 24 hours after the first daily intake) on day 21 of cycle 1 (the day before vinorelbine administration) and on the following day (day 1 of cycle 2) after vinorelbine administration: time, plus 48 and 72 hours after the first sorafenib intake on cycle 2 day 1.

## Results

**Fig 1:**
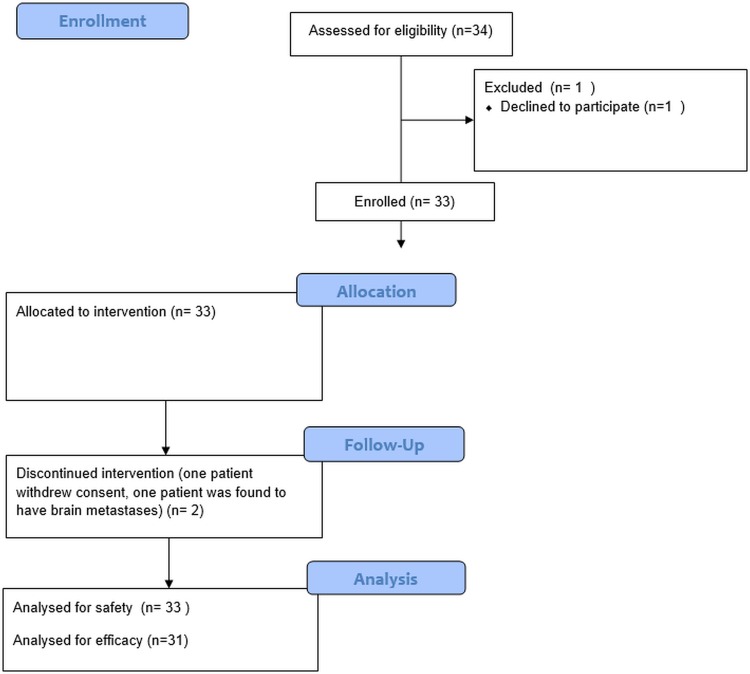
Flow diagram of the patients enrolled in the study.

Between Dec 2007 and July 2011, 34 patients were screened and 33 were consented to participate in the study (Fig 1). Two patients discontinued treatment during the first cycle of treatment in the absence of DLT, one because of the discovery of brain metastases and the other one withdrew consent. These two patients were then deemed not evaluable for efficacy analysis, but were included in the safety analysis. There was no loss to follow-up.

### Phase I

In the first cohort of patients (sorafenib 200 mg bid) one DLT after 1 cycle was observed in patient 2 (grade 3 pancreatitis), requiring expansion of this cohort to six patients total, with no further DLT reported at cycle 1. This dose level was confirmed to be safe also for repeated cycles: only one patient with a baseline borderline LVEF (< 55%) had to discontinue treatment after 8 cycles because of toxicity, developing a recurrent grade 2 LVEF decrease, that recovered to >50% after treatment was stopped.

In cohort 2 (sorafenib 400 mg bid) one patient out of six developed a DLT at cycle 1, consisting of a grade 4 increase in amylase and lipase. Even if this was compatible with the per-protocol definition of DLT, it was completely asymptomatic and felt to be clinically non-relevant. With repeated cycles no other patient had to discontinue treatment for DLT, but temporary drug suspensions or dose reductions were used for patients not tolerating sustained treatment. One patient decided to withdraw from the study for subjectively unacceptable grade 2 fatigue and was replaced by another evaluable patient.

The MTD was not formally reached and it was then established that the recommended dose for the phase II expansion was: vinorelbine 30 mg/m^2^ day 1,8 every 21 days and sorafenib 400 mg bid,

### Phase II: patient population

In the phase II part of the study 21 more patients started on treatment at the MTD, so as to have a total of 27 evaluable patients treated at this dose (including the 6 evaluable patients treated in the phase I cohort 2). Patients were all female, with a median age of 57 (range 35–71). They received a median of 8 cycles (1–28). Patient characteristics are summarized in Table 1.

**Table 1:** Baseline characteristics of the patients treated with the VS combination at the MTD.

Characteristics	Value
Total patients	27
Median age (range)	57 (35–71)
Median number of cycles	8 (1–28)
Characteristic	Patients, no.
Hormone status	
ER+/PR+	17
ER+/PR-	3
ER-/PR-	7
Tumor subtype	
Ductal	21
Lobular	3
Inflammatory–NOS	2
Medullary	1
Number of metastatic sites	
1	6
2	10
≥3	11
Metastatic sites	
Lung	6
Liver	13
Bone	16
Node	7
Skin	5

### Phase II: toxicity

One patient experienced a toxic death at cycle 1, with febrile neutropenia complicated with shock before she was started on G-CSF. With repeated cycles 52% of patients required at least 1 dose reduction of either drug. One patient discontinued therapy for toxicity (sustained grade 3 fatigue). Other toxicities are listed in Table 2.

**Table 2:** Adverse events observed in the 27 patients treated at the recommended dose.

GRADE	1	2	3	4	5
*Febrile neutropenia*				2 (7%)	1 (4%)
*Uncomplicated neutropenia*	5 (18%)		5 (19%)	10 (37%)	
*Fatigue*	13(48%)		5 (19%)		
*Hand-foot syndrome*	7 (26%)		3 (11%)		
*Diarrhea*	13 (48%)		1 (4%)		
*Rash*	9 (34%)		1 (4%)		
*Enteritis*			1 (4%)		
*Hypertension*			1 (4%)		
*Decrease LVEF*		1 (4%)			
Lipase / amylase*	5 (19%)		2 (7%)		
Bilirubin / GGT*	3 (11%)		3 (11%)		
*Alopecia*	6 (22%)	5 (19%)			
*Sensorial neuropathy*	5 (19%)	2 (7%)			

*Asymptomatic increase

- **Other adverse events max. grade 1–2, reported in 15–27% of patients:** *asymptomatic AST/ALT increase*, *asymptomatic ALK increase*, *mucositis*, *nausea*, *vomiting*, *diffuse pain*, *abdominal pain*, *dyspepsia*, *constipation*.
- **Other adverse events max. grade 1–2, reported in 4–11% of patients:** *thrombocytopenia*, *anemia*, *insomnia*, *depressed mood*, *weight loss*, *dyspnea*, *dysgeusia*, *increased creatinine*, *increased uric acid*, *headache*, *chills*, *myalgia*, *abscess*, *tinnitus*, *chest pain*, *hypothyroidism*, *dry mucosae*, *dry skin*.

### Phase II: efficacy

**Fig 2:**
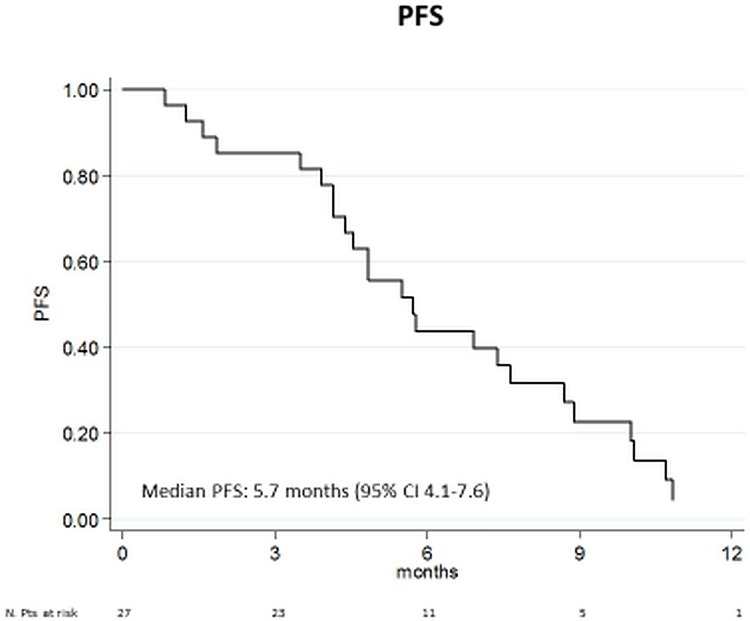
Progression-free survival of the VS combination

Best responses were as follows: 30% partial response, 55% stable disease ≥ 4 cycles, and 15% disease progression (including toxic death). Clinical benefit rate (defined as the absence of disease progression for at least 6 months) was 48%. Median progression-free survival was 5.7 months (95% CI 4.4–7.6) (Fig 2). Median time-to-progression was the same: 5.7 months (95% CI 4.5–8.7).

### Exploratory analysis

We noticed a longer PFS in the patients treated in the cohort 1 of the phase I. We then ran an unplanned hypothesis-generating analysis comparing PFS between patients started on treatment at 400 mg bid of sorafenib and maintaining this dose during treatment versus those treated at 200 mg bid from the start or reducing their dose to 200 mg bid within the first 2 cycles.

**Fig 3:**
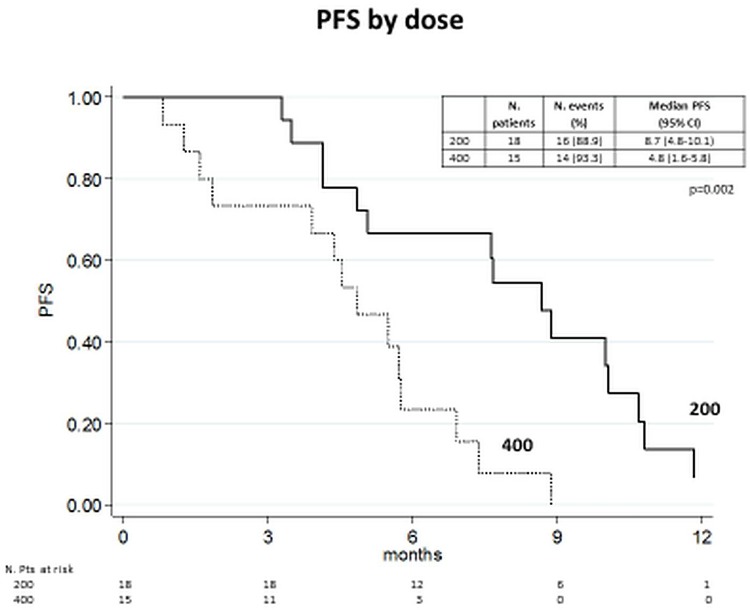
Progression-free survival by the dose of the regorafenib

There appears to be an advantage associated with the lower dose of sorafenib, with a PFS of 8.7 months in 18 patients treated at the lower dose versus 4.8 months in those treated at full doses of sorafenib (p = 0.002) (Fig 3). Results were similar when considering in the "200 mg subgroup" only the patients treated from the start at the lower dose or those requiring dose reduction because of toxicity.

### Pharmacokinetics

One of the six patients analyzed for PK analyses had to reduce the dose of sorafenib for toxicity after the first cycle, so in her case all PK analyses were re-run for treatment with 200 mg bid of sorafenib.

As sorafenib intake was every 12 hours, concentration curves for sorafenib and M2 metabolite were truncated at the 7-hour time point, in order to increase the sensitivity in the PK analysis in between doses.

Plasma levels of vinorelbine showed little interpatient variability and were compatible with a triphasic elimination profile, as described in the literature.

**Fig 4:**
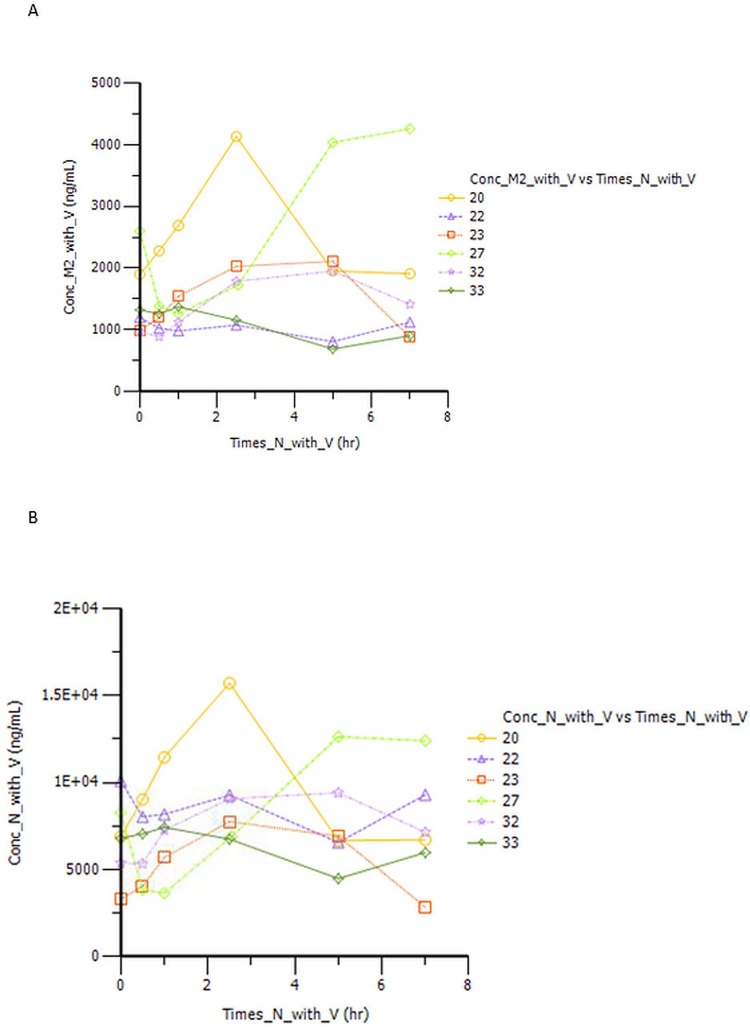
A: Plasma concentration of Sorafenib over time. B: Plasma concentration of sorafenib metabolites (M2) over time

Plasma concentrations of sorafenib (Fig 4A) and M2 metabolite (Fig 4B) show noticeable variability across the 6 patients analyzed, here in the presence of vinorelbine. Similar variability was observed also in the absence of vinorelbine (data not shown).

Table 3 summarizes mean PK parameters (in all 6 patients) with SD.

**Table 3:** Mean pharmacokinetic parameters in all 6 patients with SD.

			t½ (hr)	Cmax (ng/mL)		AUClast (hr*ng/mL)	
**V**	V alone	Mean	0.21	1301	p = 0.031	856	p = 0.219
		SD	0.04	553		358	
	V + S	Mean	0.19	2039		1057	
		SD	0.04	383		189	
**S**	S alone	Mean	1.67	9,135	p = 0.156	44800	p = 0.093
		SD	1.29	1624		3839	
	S + V	Mean	2.67	10,502		55413	
		SD	2.04	3159		11234	
**M2**	S alone	Mean	1.76	2,224	p = 0.312	10662	p = 0.312
		SD	1.18	988		4054	
	S + V	Mean	3.42	2,503		12576	
		SD	2.69	1352		5461	

V = vinorelbine S = sorafenib M2 = M2 metabolite p = significance of Wilcoxon signed-rank test

**Fig 5:**
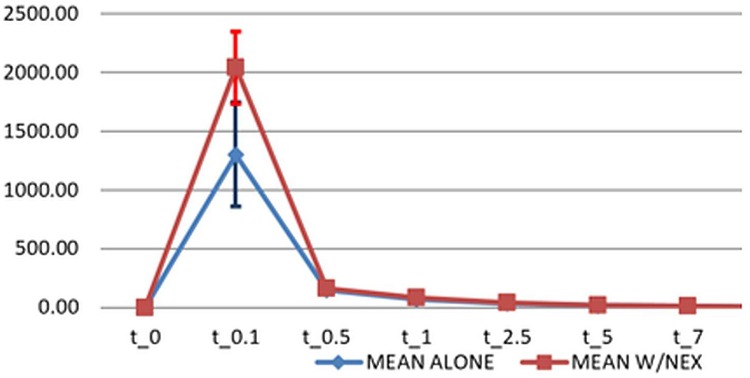
Mean plasma concentrations of vinorelbine when administered as a single agent (cycle 1 day 1, blue curve) or in the presence of steady-state concentrations of sorafenib (cycle 2 day 1, red curve).

We observe a significantly higher Cmax of vinorelbine when the drug is administered in the presence of steady-state concentrations of sorafenib (Fig 5). This was clear in 5 out of 6 patients analyzed and found to be statistically significant (both with Wilcoxon signed-rank test and with paired t-test).

There was a marginal significance for an increase of the area under the curve until the last measurable concentration (AUClast) of sorafenib in the presence of vinorelbine.

## Discussion

Antiangiogenesis therapy for MBC has been the subject of many preclinical and large randomized clinical trials with potentially controversial results. The most widely studied agent is bevacizumab, a humanized monoclonal antibody against VEGF. In a phase III randomized trial, the addition of bevacizumab to paclitaxel significantly improved PFS (median 11.8 vs 5.9 months) and increased overall response rate (ORR 36.9% vs 21.2%), without improving OS (median 26.7 vs 25.2 months) [8]. Additional trials combining bevacizumab with multiple other chemotherapy agents reported very similar results [9,10,11]. A meta-analysis of these trials confirmed that bevacizumab combined with chemotherapy in the first-line treatment of MBC significantly improves ORR and PFS, but at the expense of increased rates of grade 3–4 toxicities, and without any statistically significant OS benefit [12].

As part of the Trials to Investigate the Efficacy of Sorafenib (TIES) in breast cancer program, sorafenib has been studied with various agents in MBC in four randomized, placebo-controlled, double-blinded trials: a phase IIB of sorafenib in combination with first- or second-line capecitabine in HER2- MBC (SOLTI-0701) that showed a significant improvement in PFS of 6.4 months vs 4.1 months [13], a combination with first line paclitaxel (NU07B1) that showed a PFS of 6.9 months vs 5.6 months [14], a first- or second-line trial in combination with capecitabine or gemcitabine (AC01B07) that showed a PFS of 3.4 months vs 2.7 months [15], and a first line combination with letrozole and/or docetaxel (FM-B07-01) trial that showed PFS of 9.2 months vs. 10.2 months [16]. The SOLTI-0701 trial of sorafenib in combination with capecitabine 1000 mg/m^2^ 14 days of a 21-day cycle is the only trial that reported a statistically significant PFS improvement, without a significant impact on OS (22.2 vs 20.9 months) or overall response (38% vs 31%). To further investigate this combination, a phase III trial of sorafenib in combination with capecitabine (RESILIENCE- NCT01234337) is currently under way.

Our results from this phase I/II trial also fail to report a response rate higher than historical control data matched for line of treatment (first line only), with our reported ORR of 30% in this study being lower than the historical control of 43%. Nevertheless, the PFS observed in this study is still interesting, with a clinical benefit >6 months in 16 of 33 patients (48%). Acting mainly as a cytostatic agent, sorafenib might contribute more to a longer disease control rather than increasing tumor shrinkage. This highlights the challenges involved in exploring combinations with biological agents in small-sized phase 2 trials, in particular for what concerns the selection of the ideal primary endpoint and the importance to also develop pharmacodynamics markers of biological activity.

Recently, another phase I/II trial in MBC reported on the combination of vinorelbine and sorafenib [17]. With 44% patients free of progression at 4 months, authors concluded that the VS regimen did not appear clearly superior to historical data for single-agent vinorelbine, although we would point out that a reliable comparison is almost impossible given that patients were treated in this study in a combination of first-, second- and third-line therapies. Other characteristics of that trial distinguishing it from ours and making its interpretation somehow more complex include the fact that 41% of patients had triple negative disease, and 24% of patients had received bevacizumab as part of previous treatments for MBC.

Another significant finding in our study is that although the safety analysis after 1 cycle failed to determine a toxic dose level, with repeated cycles about half of the patients required at least one dose reduction of either drug. In most cases this was done to optimize treatment tolerability in the absence of life-threatening toxicities, although we also observed one case of fatal febrile neutropenia. Unexpected low-grade toxicity included a significant portion of patients with grade 1–2 hair loss, which is usually not observed when either drug is used as a single agent.

We believe that part of these findings derive from the significant pharmacokinetic interaction between sorafenib and vinorelbine that we observed, resulting in a marginally significant increase of AUClast of sorafenib and more importantly a statistically significant increase in Cmax of vinorelbine when the two drugs are combined. Considering all of the above, a reasonable option to pursue development of the VS combination in clinical practice with better tolerability would be to start therapy with sorafenib at 200 mg bid and full doses of vinorelbine, considering a gradual dose increase to full doses for those patients in which no grade 2–3 toxicity is observed. It is somehow reassuring that we observed no detrimental effect for patients receiving sorafenib at the reduced dose of 200 mg bid.

## Conclusion

Combining sorafenib and vinorelbine at full doses is feasible, but not devoid of toxicity for repeated cycles, most likely due to a significant PK interaction. Prolonged disease control is observed also with a reduced dose of sorafenib. This combination should be only pursued if valid pharmacodynamics biomarkers or predictive biomarkers become available.

## Supporting Information

S1 File Nexavar Protocol. (PDF)

S2 File TREND Statement (PDF)
