# Supplementary material for: Phase I/II Trial of Sorafenib in Combination with Vinorelbine as First-Line Chemotherapy for Metastatic Breast Cancer
Source: PLoS One. 2016 Dec 19;11(12):e0167906. doi: 10.1371/journal.pone.0167906 (PMC5167316; doi:10.1371/journal.pone.0167906)
Supplement: S1 File — (PDF) [file pone.0167906.s001.pdf]

A Phase Ib/II trial of combination of vinorelbine with sorafenib (BAY 43-9006) as first-line treatment in patients with metastatic breast cancer

Principal Investigator:
Dr. Lawrence Panasci
lpanasci@hotmail.com
514-340-8248

Co- Investigator:
Dr. Cristiano Ferrario
cristianoferrario@gmail.com
514-340-8222 local 2782

McGill Department of Oncology
Including:
Jewish General Hospital
3755 Cote Ste Catherine
Montreal, Quebec, Canada
H3T1E2
Fax: 514-398-2754 and 514-398-8302

Supported by:
Bayer Inc.
Bayer Healthcare Pharmaceuticals
77 Belfield Road
Toronto, Ontario
M9W 1G6

Version date: March 8th, 2007

TABLE OF CONTENTS

**1.**	**Background**	**5**
**2.**	**Rationale for the use of vinorelbine**	**5**
2.1	Pharmacokinetics of vinorelbine	7
2.2	Toxicity of vinorelbine	8
**3.**	**Rationale for the use of sorafenib**	**8**
3.1	Pharmacokinetics of sorafenib	11
3.2	Toxicity of sorafenib	11
**4.**	**Rationale for the combination of sorafenib and vinorelbine**	**12**
**5.**	**Trial objectives**	**15**
5.1	Primary objectives	15
5.2	Secondary objectives	15
**6.**	**Study design**	**15**
6.1	Enrollment procedures	16
**7.**	**Inclusion criteria**	**17**
**8.**	**Exclusion criteria**	**17**
**9.**	**Therapy administration**	**18**
9.1	Phase I	18
9.2	Phase II	19
9.3	Vinorelbine administration	19
9.4	Sorafenib administration	19
9.5	Duration of therapy	19
**10.**	**Patient assessment**	**20**
10.1	Screening procedures	20
10.2	Monitoring of patients treated with combination therapy	20
10.3	Monitoring of patients entering monotherapy with sorafenib	21
**11.**	**Toxicity and dose delays - modifications**	**23**
11.1	Criteria for retreatment	23
11.2	Hematological toxicity	23
11.3	Diarrhea	24
11.4	Hand-foot skin syndrome (HFS)	24

11.5 Skin rash 25
11.6 Hypertension 25
11.7 Cardiac toxicity 25
11.8 Other non-hematological toxicities 26
11.9 Dose modifications 26
12. Definition of dose-limiting toxicity (DLT) 27
13. Concomitant medications 27
13.1 Anticancer treatments 27
13.2 Surgical procedures 28
13.3 Allowed medications 28
13.4 Hematopoietic growth factors 28
14. Discontinuation of therapy and withdrawal from study 29
15. Assessment and reporting of adverse events 29
15.1 Definition of Adverse Event (AE) 29
15.2 Assessment of adverse event severity and relationship to treatment 30
15.3 Monitoring adverse events 30
15.4 Definition of Serious Adverse Event (SAE) 31
15.5 Report of Serious Adverse Events (SAEs) 32
15.6 Reporting of a subject's death 32
15.7 Report of pregnancies 33
16. Statistical considerations 34
16.1 Sample size justification for Phase II 34
16.2 Definition of study outcomes 35
16.3 Analysis populations 35
16.4 Analysis of primary endpoints 36
16.5 Analysis of event-free times 36
16.6 Safety analysis 36
17. Pharmacokinetics (PK) analysis 37
17.1 PK analysis of vinorelbine 37
17.2 PK analysis of sorafenib 37
18. Data handling and record keeping 39

18.1 Case Report Forms (CRFs) 39
18.2 Record retention 39
19. Ethics **39**
19.1 Institutional Review Board (IRB)/Independent Ethics Committee (IEC) 39
19.2 Ethical conduct of the trial 40
19.3 Subject information and consent 40
20. Publication policy **40**
21. References **42**
Appendix A Declaration of Helsinki **46**
Appendix B ECOG Performance Status **49**
Appendix C Creatinine clearance **50**
Appendix D NCI Common Toxicity Criteria **51**
Appendix E RECIST criteria **52**
Appendix F Instructions for handling of PK samples **57**

PART I: BACKGROUND AND RATIONALE

1. BACKGROUND

Breast cancer is the most common malignancy diagnosed in women. While the incidence of breast cancer is increasing, the mortality has decreased significantly over the last ten years, possibly secondary to earlier diagnosis and the use of adjuvant hormonal/chemotherapy. When breast cancer metastasizes to distant organs, the disease is typically incurable although long-term remissions ($\geq$ 10 years) on hormonal or chemotherapy occur in a small percentage (1-5%) of patients. Chemotherapy, including classical CMF, taxanes, and anthracyclines, produces objective responses in 40-60% of patients. Combination chemotherapy with anthracyclines and/or taxanes produces a higher response rate that CMF with a questionable effect on survival, probably because sequential therapy (ie. adriamycin and/or taxanes) produces responses which negate the effect of using these agents as first line therapy[1].

On the other hand, second line therapy with docetaxel as compared to mitomycin C/vinblastine results in a higher response rate and overall median survival (11.4 months versus 8.7 months). While these results are significant, it must be emphasized that the difference in median survival is less than 3 months, with a similar quality of life[2].

Trastuzumab plus adriamycin/cyclophosphamide chemotherapy in patients over-expressing HER2 in their tumors (less than 20% of metastatic breast cancer patients will benefit) shows a clear median survival advantage (over 5 months), but significantly more cardiotoxicity than adriamycin/cytoxan chemotherapy alone. The result in this subset of metastatic breast cancer patients represents one of the few reports of a significant median survival advantage over adriamycin/cytoxan chemotherapy[3].

For instance, in a recent randomized trial of FAC vs TAC in metastatic breast cancer, there was no difference in survival or time to progression probable because of sequential use of docetaxel in the FAC group[4].

Metastatic breast cancer is a virtually incurable disease, for which achieving an objective response to therapies has a questionable effect on survival, and, in many cases, aggressive treatment is not justified. Rather, there is a need to develop and assess new combinations that might maintain a good efficacy profile, without significantly compromising patients' quality of life.

2. RATIONALE FOR THE USE OF VINORELBINE

Vinorelbine is a semi-synthetic vinca-alkaloid, derived from *vinca rosea*. Vinca alkaloids

are structurally similar compounds comprised of two multiringed units, vindoline and catharanthine. Vinorelbine differs from the natural compound by the presence of an eight-member catharanthine ring, site of structural modification.
Vinorelbine has activity in a broad spectrum of tumors, as a mitotic spindle poison that impairs chromosomal segregation during mitosis and blocks cells at G2/M, at concentrations close to the IC_{50}[5], between 1 and 50 nM[6]. Microtubules are the principal target of vinorelbine[7], as the eight-member catharanthine ring forms covalent reversible bonds with tubulin, the monomeric subunit of microtubules. Interestingly, vinorelbine shows a preferential binding to mitotic rather than axonal microtubules, possibly determining its tumor selectivity and low neurotoxicity[8,9].
Disorganization of the microtubule structure, through induction of p53 and activation/inactivation of several protein kinases[10], eventually triggers apoptosis[11].

In metastatic breast cancer single-agent vinorelbine shows activity equivalent to many standard agents, including anthracyclines, with a response rate for first line treatment of 35-60%, documented by several phase II trials worldwide[12-18].

In the largest phase II trial on single-agent vinorelbine as first line treatment for metastatic breast cancer, 145 women received 30 mg/m^2 weekly[12]. The overall response rate was 41%, and 30% of patients had stable disease. The median time to treatment failure was 6 months, and median survival is 18 months.
Previous therapy with anthracyclines or CMF regimen does not appear to have any influence on response[12,15].

Similar response rates (50%) are observed with vinorelbine at 25 mg/m^2 weekly[17], or in elderly patients[13,19], for whom no need of dose-reduction is reported.
Vinorelbine can also be administered on days 1 and 8 of 21-day cycles, to better fit the two main treatment periodicities (corresponding to one week and three weeks), with no decrease in the administered dose intensity[15].

A randomized phase II study in 183 pretreated anthracycline-refractory patients compared weekly vinorelbine to 3-weekly melphalan. The trial demonstrated improved time to progression (12 vs 8 weeks, $p<0.001$) and improved 1-year survival (35.7% vs 21.7%, $p=0.034$) for patients treated with vinorelbine[20].

Interestingly, a Canadian cost-benefit retrospective analysis compared first-line single agents vinorelbine, paclitaxel or docetaxel in 88 anthracycline pretreated patients with metastatic breast cancer. Vinorelbine appears to provide at least equivalent quality-adjusted progression-free survival, with a relevant economic advantage related to its lower drug acquisition cost and better toxicity profile[21].

As a consequence of its good tolerability, vinorelbine has been tested in combinations with all the other major chemotherapy agents for breast cancer: anthracyclines[22,23], mitoxantrone[24], 5-flurouracil/capecitabine[25,26], taxanes[27,28], platinum salts[29], gemcitabine[30], showing good activity without significant increase in toxicity.

A combination of vinorelbine with other chemotherapy agents can be associated in some reports with relatively high response rates[26,28]. Nevertheless, no randomized trial to date has shown superiority in survival for any of these combinations, when compared to sequential therapy with the same agents[31].

The only exception, though formally not proven, likely concerns vinorelbine combination with trastuzumab in HER-2 positive metastatic breast cancer.
The high efficacy of this combination was predicted by *in vitro* studies on HER2-positive cell lines, demonstrating a more powerful synergism than trastuzumab combination with taxanes[32].
The clinical feasibility and efficacy of this combination is highlighted by several phase II trials in metastatic breast cancer[33-35].
The addition of trastuzumab to vinorelbine in these patients eventually confers a prognosis even better than HER-2 negative tumors, possibly "reversing" the natural history of the poorer prognosis of HER-2 overexpressing tumors[36].

2.1 Pharmacokinetics of vinorelbine

Similar to other antitumor Vinca alkaloids, the pharmacokinetic properties of vinorelbine can be described by a three-compartment model[37], with plasma concentration decaying in a triphasic manner after brief intravenous infusion.

The initial rapid decline primarily represents drug distribution to the peripheral compartments: vinorelbine diffuses freely into tissues, showing a large volume of distribution. After a dose of 30 mg/m^2 i.v., an initial high peak of 5 mmol rapidly decays to about 1 mmol at 2 hours. Distribution in blood is rapid, with binding of 78% of the drug to platelets and lymphocytes and a further 13.5% to plasma proteins, and only 1.7% left as free drug in the first 2 hours after administration. Subsequently, binding to plasma proteins is in order of 70-80%. In experimental animals, within 30 min of administration, vinorelbine is highly concentrated in bile, excretory organs (spleen, liver, kidney), lung, muscle and heart[38]. High levels of drug are found in both normal lung and tumor tissue, and diffusion out of tumor tissue is slow[39]. Brain and plasma levels are comparable in animal studies[38]. In pregnant rats, vinorelbine crosses the placenta and is detectable in the fetus.

The "second phase" of plasma concentration represents the metabolism and excretion of the drug. After metabolic processing by hepatic cytochrome P450 isoenzymes in the CYP3A subfamily[40], vinorelbine undergoes substantial hepatic elimination in humans, with large amounts documented in feces. Two metabolites of vinorelbine have been identified in human blood, plasma, and urine: vinorelbine N-oxide and deacetylvinorelbine, the latter being the primary metabolite in humans, theoretically with antitumor activity similar to vinorelbine. Nevertheless, therapeutic doses of vinorelbine (30 mg/m^2) yield very small, if any, quantifiable levels of either metabolite in blood or urine.
The prolonged terminal phase is due to relatively slow efflux of vinorelbine from

peripheral compartments. The terminal phase half-life averages 27.7 to 43.6 hours and the mean plasma clearance ranges from 0.97 to 1.26 L/hr/kg. Steady-state volume of distribution (V_{ss}) values range from 25.4 to 40.1 L/kg[41].

Few analyses of repeated infusions of vinorelbine suggest a general decrease in plasma concentrations, without significant modifications of pharmacokinetic parameters[37,41].

2.2 Toxicity of vinorelbine

The majority of data available for vinorelbine are based upon i.v. administration, that is generally well-tolerated.

Dose-limiting toxicity is leucopenia/neutropenia, reported as Grade 3-4 in 14-52% of patients treated with a weekly schedule. It is reversible, lasting 7-14 days, not cumulative[42]. Thrombocytopenia is rare during therapy with vinorelbine.

Peripheral neuropathy, less frequent than with other vinca-alkaloids, has been reported in up to 30% of patients treated with vinorelbine, but there was Grade 3-4 neuropathy only in 1% of cases[43]. Neurotoxic effects include decreased deep-tendon reflexes, constipation, parasthesiae, myalgia and paralytic ileus.

Severe nausea and vomiting are relatively infrequent with vinorelbine, with Grade 3-4 toxicity reported in only 1-3% of patients[42,44].

Alopecia affects only 10% of patients treated with vinorelbine and is of minor severity, so that very few patients require a wig.

Grade 3-4 cutaneous or venous reactions, e.g. pain on injection, venous pain and thrombophlebitis, have occurred in 5-10% of patients receiving vinorelbine[45]. This can to some extent be prevented by adequate flushing through of the vein with normal saline following injection of the drug. In patients with difficult peripheral venous access, a central venous administration is preferable.

3. RATIONALE FOR THE USE OF SORAFENIB

The chemical name of BAY 43-9006 is *N*-(3-trifluoromethyl-4-chlorophenyl)-*N*'-(4-(2-methylcarbamoyl pyridin-4-yl)oxyphenyl)urea, and the structural formula is shown below:

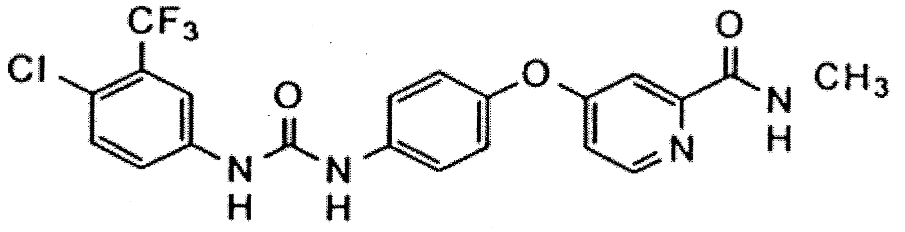

Sorafenib (BAY 43-9006) was originally identified by screening of small molecules inhibiting C-Raf kinase, and found to be a potent competitive inhibitor of ATP binding to C-Raf (Raf-1), wild-type B-Raf and mutant B-Raf (^{V599E}B-Raf), in all cases with an IC_{50} in the low nanomolar range[46].

The mammalian *raf* family consists of three genes: *A-raf*, *B-raf* and *C-raf* , encoding for the cytoplasmic proteins A-Raf, B-Raf and C-Raf (or Raf-1)[47].
The Raf serine/threonine kinases are the main effectors of Ras in the mitogen-activated protein kinase (MAPK) pathway. Activated (phosphorilated) Raf principally propagates signaling by phosphorylating MEK1 and MEK2[48]. This pathway regulates fundamental cellular functions in cancer, such as proliferation, survival, transformation, and metastasis. Furthermore, C-Raf is also capable of activating other signaling elements independent of MAPK pathway, such as NF-κB[49], Bcl-2[50] and the multi-drug resistance gene mdr-1[51].

In clinical specimens, C-Raf is detectable in the cytosol of breast cancer cells and not in the normal counterpart[52]. Baseline expression of phosphorylated (i.e. activated) C-Raf is elevated at least in some breast cancer cell lines[53].

In several human cancers, somatic mutations in the *B-Raf* gene (less frequently in *C-Raf*) are reported, with a particularly high incidence of activating missense mutations in malignant melanoma (66%), and a much lower frequency (2%) in breast cancer cell lines[54].

In breast cancer, the activation of the RAS-RAF-MEK-ERK-MAP kinase cascade is mainly due to stimulation by cytoplasmic or membrane receptors, such as tyrosine-kinases EGFR and HER-2[55], whose ligand-dependent dimerization is associated with C-Raf translocation and transient accumulation at the plasma membrane.
Moreover, estrogen stimulation of ER+ breast cancer cells can induce C-Raf and A-Raf activities[56].
Treatment of breast cancer xenografts with agents targeting C-Raf (antisense oligonucleotide[57], siRNA[58]) inhibits tumor growth in *in vivo* models.

Interestingly, further characterization of sorafenib showed potent inhibition also of a defined spectrum of receptor tyrosine kinases (RTKs) mainly involved in angiogenesis: human VEGFR2, Flt-3, platelet-derived growth factor receptor beta (PDGFR-β) c-kit, FGFR-1, as well as p38α, a member of the MAPK family[59].

Table 1.
Mean IC_{50} (nmol/L) of sorafenib for several human proteins, assessed in biochemical assays[59].

		IC_{50}
C-Raf	*Activator of MAPK pathway*	6
Wild type B-Raf	*Activator of MAPK pathway*	22
^{V599E}B-Raf mutant	*Constitutively active variant*	38
VEGFR2	*Main VEGF receptor in endothelial cells*	90
Mouse PDGFR-β	*Involved in angiogenesis (perycites) and CML*	57
Flt-3	*Receptor involved in hematopoiesis/leukemia*	58
c-KIT	*Receptor in marrow progenitors, involved in GISTs, LMA*	68
FGFR-1	*Angiogenesis-related FGF receptor*	580
Others	*ERK-1, MEK-1, EGFR, HER-2, IGFR-1, PKCγ*	>10,000

The clinical relevance of angiogenesis in breast cancer[60] was highlighted by recent therapeutic success in the use of the humanized antibody bevacizumab (Avastin™)[61], which targets vascular endothelial growth factor (VEGF), the major mediator of angiogenesis in tumors. Indeed, VEGF is responsible for the activation of endothelial cells that initiates the angiogenic process[62].

In blood vessel endothelial cells, the main VEGF receptor is considered to be the tyrosine kinase VEGF receptor 2 (VEGFR2, or kinase insert domain receptor, KDR). VEGFR2 activation by VEGF mediates endothelial proliferation and motility, tube formation and vascular permeability[63,64].

Interestingly, also VEGFR2 is known to transduce downstream signals in part through C-Raf, acting as a major survival regulator in endothelial cells too[65]. So, sorafenib could efficiently target endothelial cells activation through a blockage of this pathway at two different points.

Sorafenib is then expected to combine a targeted antiproliferative effect on tumor cells together with antiangiogenesis properties.

Indeed, in cellular assays, sorafenib reduces basal phosphorylation of the MAPK pathway in a panel of breast and other cancer cell lines expressing either mutant K-RAS, mutant B-Raf, or wild-type Ras or Raf; the inhibition of VEGFR2, Flt-3 and PDGFR-β phosphorylation is also confirmed[59].

In nude mice bearing human cancer xenografts, sorafenib treatment results in high levels of tumor growth inhibition, with maximum effect on the breast cancer xenograft with MDA-MB-231, a cell line bearing both K-ras and B-raf mutations. Notably, sorafenib shows growth inhibition also in Colo-205 xenografts, where no inhibition of the MAPK pathway is clearly documented in cancer cells *in vitro*, probably as a consequence of the dramatic decrease in tumor neovascularization *in vivo*.

Several Phase I trials have been conducted with sorafenib monotherapy, to carefully evaluate the most favorable schedule of administration[66-69]. In the end, 400 mg twice daily on a continuous schedule is recommended for Phase II/III trials[69]. Early clinical trials suggest that sorafenib acts as a cytostatic agent: approximately 50% of patients with colorectal, ovarian, hepatocellular, renal, and breast carcinoma treated in phase I studies, have stable disease as their best response[70]. Also in renal cancer, where sorafenib shows significant improvement in progression-free survival versus placebo, clinical benefit mainly consists in the induction of disease stabilization, rather then tumor shrinkage[71].

A preliminary phase II trial[72] with sorafenib in monotherapy has been conducted in 54 metastatic breast cancer patients, generally heavily pretreated: 64% have received at least four prior chemotherapy regimens. Nevertheless, 22% of the patients present a stable disease lasting more than 16 weeks; one partial response is also reported. Given the overall good tolerability of sorafenib, these results demand further assessment of its activity in breast cancer, possibly in more precocious stages.

3.1 Pharmacokinetics of sorafenib

Pharmacokinetic studies in rodents and dogs have demonstrated that sorafenib clearance is much lower than normal liver plasma flow. Its low steady-state volume of distribution (approximately 0.7 to 0.93 L/Kg) suggests that tissue affinity is low and plasma protein binding is high, with a mean free fraction of 1.2% in human to 2.5% in mouse.

The pharmacokinetics in mice are dose proportional over a biologically relevant dosing range, attaining tissue concentrations that are several fold higher than IC_{50} values *in vitro*[59,73]. In rodents, oral bioavailability is high (approximately 79%). At higher doses, drug exposure increases disproportionately, possibly for saturation of gastrointestinal absorption.

In animals, drug disposition is mainly through CYP3A4 metabolism, followed by biliary and fecal excretion (approximately 90%). *In vitro* metabolism studies in human systems confirm extensive metabolism by CYP3A4, and also early clinical data indicate hepatic metabolism and fecal excretion.

Pharmacokinetic studies in humans confirm dose proportionality up to 600 mg twice daily. The drug is absorbed slowly and at a moderate level after the first dose of tablet formulation, and C_{max} occurs at 2.5 to 12.5 hours after administration.
AUC and C_{max} values demonstrate high inter-patient variability following single oral doses of BAY 43-9006. Intake of food before dosing has no relevant pharmacokinetic impact, except for slightly prolonging t_{max}. Mean $t_{1/2}$ ranges from 24 to 38 hours.
Substantial accumulation in plasma is observed after multiple bid administrations[69], again with highly variable AUC and C_{max}.
Steady state is achieved by 7 days[74,75], and terminal half-life values ranges from 30 to 45 hours.

Preliminary data indicate that BAY 43-9006 is subjected to entero-hepatic circulation, the extent of which may vary in different patients.

3.2. Toxicity of sorafenib

Sorafenib monotherapy is generally well tolerated, with few side effects.
The main toxicities reported at 400 mg twice daily continuously are gastrointestinal (~60%) and dermatologic (~40%) in nature[69,71,72,76].

Diarrhea, the most common gastrointestinal adverse event (~20%), in most cases is mild to moderate in severity, being reported of grade 3 in 4-8% of patients. Grade 3 diarrhea typically resolves within 24 to 48 hours of drug withdrawal, while G1-2 diarrhea is easily managed with oral loperamide, without drug withdrawal.

Hand-foot syndrome (HFS) is reported in ~60% of patients, being Grade 3 in 6-13% of patients. It manifests with digital desquamation and discomfort or painful symmetrical erythema/edema of palms and soles, often preceded or accompanied by paraesthesias. This toxicity is reversible with temporary drug suspension, and restoration of full doses is possible after its resolution[77].

Cutaneous rash (typically a facial/scalp erythema) is also frequent (32%-62%), of moderate severity in 6%-10% of cases. Grade 1-2 pruritus is reported in 22% of cases. Alopecia is a rare event.

Fatigue is common (39-56%), but of Grade 3 in only 6% of patients.

Grade 3/4 hypertension seems to occur with higher incidence in renal cancer patients (24% of the cases), while in other cancers is seldom reported as Grade 3.

Other less common toxicities include nausea (G2 in 4%), anorexia (28%, G2 in 4%), stomatitis (G2 in 4%), serum amylase/lipase increase (4% G3, reversible upon withdrawal of the drug within 2 weeks), serum bilirubin increase, lymphopenia and anemia.

Interestingly, in advanced solid tumors treated with sorafenib, patients reporting skin toxicity or diarrhea have a significantly longer time to progression[76].

4. RATIONALE FOR THE COMBINATION OF SORAFENIB AND VINORELBINE

The idea of combining vinorelbine and sorafenib in metastatic breast cancer patients is particularly intriguing, both for the lack of severe toxicity of either drugs, and for a possible synergism, given by a "chemosensitizing" function of sorafenib.

Several phase Ib/II trials have evaluated the feasibility of administering sorafenib with various other agents, in most cases without evidence of unexpected toxicity with drugs at full doses[78,79]. Pharmacokinetic interactions between sorafenib and these drugs were not reported.

In a phase II trial of combination with gemcitabine a relatively high incidence of Grade 3-4 thrombosis/embolic events is reported (16%), but 4 out of 5 cases are reported in pancreatic cancer patients, known to be at higher risk[80]. No comparison against combination with placebo is available.

The combination of sorafenib with vinorelbine has not been tested in humans yet.

Overexpression of constitutively active C-Raf in breast cancer models is associated with chemoresistance[81]. Conversely, an efficient delivery *in vivo* of a C-Raf antisense oligonucleotide acts as a powerful chemo-sensitizer in breast tumor xenografts[82].

Favourable cytotoxic effects were reported after treatment of a broad spectrum of human cancer cell lines and xenografts, harboring both wild-type and mutated forms of ras or raf, with sorafenib and several chemotherapeutic agents, including vinorelbine, without evidence of enhanced toxicity[65,83].
The combination of antiangiogenic compounds and "classical" cytotoxic agents is potentially synergistic also as a consequence of the tumor vasculature "normalization", induced by VEGF-pathway inhibitors. Indeed, one of the main challenges with cytotoxic therapies is gaining access into the interior of the tumor, because tumor vessels are structurally and functionally abnormal, irregular, and leaky, as a consequence of the pathological activation of the angiogenic process. In fact, this causes increased interstitial fluid pressure (IFP) in most tumors and results in impaired flow of fluid, macromolecules, and oxygen to the tumor. Also tumor penetration by cytotoxic agents is consequently inhibited. Therefore, it was proposed[84] that if tumor vasculature could be "normalized" with anti-VEGF therapy, through apoptosis of tumor endothelial cells and a subsequent decrease in IFP, this would facilitate a more efficient delivery of therapeutic agents to the tumor. This hypothesis is confirmed by studies with anti-VEGF antibody bevacizumab, causing reduction in interstitial fluid pressure and increase in intratumoral uptake of chemotherapy[85]. So, it is reasonable to assume that a similar effect mediated by sorafenib might allow a more effective delivery of vinorelbine to malignant cells.

Another interesting evidence comes from studies on a variety of orthotopic human breast cancer xenografts selected for multi-drug resistance. These tumors respond in a significant and durable manner to continuous low-dose ("metronomic") chemotherapy regimens, when used in combination with an anti-VEGFR2 neutralizing antibody. In the case of a combination with tubulin inhibitors (vinblastine and paclitaxel) there is no evidence of significant toxicity[86]. Actually, vinorelbine is often administered in breast cancer treatment in a fractionated schedule, so that it is possible to apply this same schedule in the clinical setting, in combination with sorafenib as a VEGFR2-neutralizing agent.

Finally, vinorelbine is gradually emerging as a very appealing option for combinations with new targeted therapies, as highlighted by the successful combination with trastuzumab.

Indeed, an interesting phase III trial has compared first-line trastuzumab combined with either vinorelbine or a taxane in metastatic breast cancer patients, but unfortunately the trial has been prematurely closed after enrollment of only 81 of the 250 patients initially planned[87]. Patients receiving trastuzumab and vinorelbine tend to have a higher response rate (51% vs 40%) and median time to progression (8.5 vs 6 months), even though at non-statistically significant levels.

The use of vinorelbine/trastuzumab in the adjuvant setting has been tested[88] in a trial randomizing 1,010 breast cancer patients to receive three cycles of either docetaxel or vinorelbine, followed by three cycles of FEC (fluorouracil, epirubicin, cyclophosphamide). In this study, the 232 HER-2 positive patients are further randomized to receive or not to receive nine weekly trastuzumab infusions. Interestingly, even though global recurrence free survival at three years is better with docetaxel than with vinorelbine (HR 0.58; p=0.005), HER-2 positive patients treated with trastuzumab have better 3-year recurrence free survival than those who did not receive trastuzumab, independently of the type of combined chemotherapy (docetaxel of vinorelbine), again suggesting that the combination of trastuzumab with vinorelbine in the treatment of breast cancer is at least as efficient as the combination with taxanes.
In conclusion, it is worthy to test the efficacy of the combination of vinorelbine and sorafenib in patients with metastatic breast cancer.

This combination would have also the notable expected advantage of producing much less nausea/vomiting, hair loss and fatigue as compared to other classical anticancer agents (anthracyclines, taxanes and alkylating agents) utilized in the treatment of metastatic breast cancer. This should improve the quality of life and the rate of patients accepting chemotherapy treatment.

Importantly, any relevant pharmacological interaction between the two drugs will need to be ruled out, particularly as they share the same metabolic path (through hepatic isoenzymes CYP3A).

PART II: STUDY PROTOCOL

5. TRIAL OBJECTIVES

To assess the safety and efficacy of sorafenib in combination with vinorelbine, as first line treatment for metastatic breast cancer.

5.1 Primary Objectives

Phase I: To assess the optimal dose of sorafenib in combination with vinorelbine.
Phase II: To assess the clinical benefit (response rate + disease control rate) in women with metastatic breast cancer, when treated with this combination at the optimal doses.

5.2 Secondary Objectives

To assess:

- toxicity and safety;
- median time to best response;
- median duration of response;
- median progression free survival;
- overall 2 year survival in women with metastatic breast cancer, when treated with

the combination of vinorelbine and sorafenib at optimal doses.

To assess pharmacokinetic steady state parameters and interaction between vinorelbine and sorafenib when administered together at optimal doses.

6. STUDY DESIGN

This will be a non-randomized, open label, phase I/II, multi-centre study of sorafenib in combination with fixed doses of vinorelbine as first-line treatment for women with metastatic breast cancer.

In the Phase I of the study, 6-12 patients will be recruited in a single center (Jewish General Hospital, Montreal).

In the Phase II part, 21-24 more patients will be recruited and treated in six different centers in Quebec.

The first part of the study will test increasing doses of sorafenib, combined with regular administration of vinorelbine at fixed therapeutic doses (30 mg per square meter on days 1 and 8 of 21-day cycles).

Dose escalations will be made in subsequent cohorts of patients, up to the recommended dose for sorafenib monotherapy. Sorafenib doses will be: 200 mg bid (cohort 1) and 400 mg bid (cohort 2).

In the second part of the trial, the cohort of patients treated at the recommended dose will be expanded, up to a total of 27 patients treated with this schedule of the combination regimen (see Section 16.2).

Six patients treated at the recommended dose will be assessed for pharmacokinetic analysis.

Clinical follow-up, including physical examination, laboratory testing and evaluation of adverse event will be performed on day 1 and 8 of each cycle.
Patients will have appropriate radiological imaging to document baseline disease within 4 weeks prior to study entry. Radiological imaging will be repeated regularly after every two cycles, until disease progression or unacceptable toxicity, up to 8 cycles (or after at least 4 cycles after achievement of the best response obtained).

Those patients whose disease has not progressed during the combination treatment, will continue receiving sorafenib alone at the dose of 400 mg bid and will be monitored every 42 days, with clinical and radiological follow-up.

All patients will be followed for a minimum of 2 years for survival and residual toxicity.

Safety will be assessed by examination of adverse events, clinical laboratory data, and vital signs. Adverse events, use of growth factors, and changes in laboratory parameters will be summarized and tabulated. Objective response (CR, PR, SD, and PD) will be summarized and tabulated. Time to response, duration of response, time to progression, and survival will be calculated. Kaplan-Meier estimates and curves will be used to present these time-to-event data.

6.1 Enrollment Procedures

Registration will be performed after all pretreatment procedures have been completed (section 11.1) and the investigator has reviewed all inclusion and exclusion criteria (sections 8 and 9).

Registration and assignment of subject number will be accomplished by facsimile.
Subject number assignment will begin in ascending order starting and will be site-specific (i.e. site 1 gets patient numbers 10-19, site 2 gets patient numbers 20-29, etc.).

7. INCLUSION CRITERIA

Patients meeting the following criteria will be eligible for enrollment:

1. Women affected by histologically proven metastatic breast cancer.
2. Tumor not susceptible to therapy with trastuzumab, defined as FISH negative for HER-2 amplification or immunohistochemistry 0-1+ for HER-2 expression.
3. Female, age ≥ 18.
4. Documented measurable disease by appropriate radiologic imaging according to RECIST criteria (see Appendix E). Lesions in previously irradiated areas are not considered measurable disease, unless progression has been documented post-radiation.
5. ECOG performance status 0-1 (see Appendix B).
6. Life expectancy > 6 months.
7. Adequate bone marrow function, as indicated by:
- hemoglobin ≥ 90 g/L;
- neutrophils ≥ 1.5 x 10^9/L;
- platelets ≥ 100 x 10^9/L.
8. Adequate renal function, as indicated by serum creatinine ≤1.5 times the upper limit of normal and/or Creatinine Clearance calculated as >50% lower normal limit, or estimated as ≥ 50 ml/min (Appendix C).
9. Adequate liver function, as indicated by:

- bilirubin $\leq$ 1.5 times upper normal limit;
- AST and ALT $\leq$ 2 times upper normal limit.

10. Left ventricular ejection fraction (LVEF) $\geq$50% as measured by either multigated acquisition (MUGA) scan or echocardiogram (ECHO).
11. No therapy for breast cancer in the 4 weeks preceding the therapy start.
12. Women of childbearing potential must be using adequate contraception and have a negative pregnancy test at the time of enrollment.
13. Patient able to understand and give written informed consent.

8. EXCLUSION CRITERIA

Patients are excluded from the study if any of the following criteria is met:

1. Patients with locally advanced breast cancer or stage IIIb only.
2. Presence of only non-measurable disease.
3. Previous (neo)adjuvant chemotherapy with vinorelbine.
4. Any previous anti-angiogenic therapy.
5. Any previous chemotherapy for metastatic breast cancer. Previous hormonal treatments or radiotherapy for metastatic disease are allowed.
6. Radiotherapy, chemotherapy or hormonal therapy for breast cancer in the last 4 weeks prior to starting the study treatment.
7. Major surgery within 4 weeks of first study treatment, or minor surgery (including placement of an access device) within 7 days of therapy start.
8. Presence of life-threatening disease or central nervous system localizations.
9. Evidence of HER-2 positive breast cancer, defined as FISH-positive for amplification or score 3+ by immunohistochemistry.
10. Any other possibly active primary tumor, except basal cell carcinoma of the skin, or carcinoma in situ of the cervix.
11. Clinically significant hepatic disease with respect to hepatitis B, hepatitis C, cirrhosis or other liver diseases.
12. Uncontrolled bacterial, viral or fungal infection.
13. Previous history of ischemic disease.
14. Patients with previous history of thrombo-embolic events, or with documented risk factors for thrombotic disease other than cancer.
15. History of gross hemorrhage within the past 6 months (e.g., hemoptysis or hematuria requiring medical intervention).
16. Uncontrolled hypertension.
17. Other serious medical conditions, such as uncontrolled cardiac disease, severe pulmonary disease, uncontrolled diabetes.
18. Patient exhibiting confusion or disorientation.
19. Any condition (medical, social, psychological, geographical) that would prevent adequate follow-up.
20. Patient is pregnant, or is breast-feeding, or is unwilling to use adequate contraception.
21. Failure to give informed consent.

9. THERAPY ADMINISTRATION

9.1 Phase I

In the first part of the study patients will test increasing doses of sorafenib, combined with a fixed schedule of vinorelbine.
Vinorelbine will be administered at the dose of 30 mg per square meter on days 1 and 8 of 21-day cycles.
Dose escalations will be made only for sorafenib, in subsequent cohorts of patients, up to the recommended dose for sorafenib monotherapy.
Planned dose levels for sorafenib are: 200 mg bid (cohort 1) and 400 mg bid (cohort 2).

Three patients will be enrolled at the first dose level. In the absence of a dose-limiting toxicity at the end of the first cycle, three patients will be enrolled in the second dose level. If any patient experiences a dose-limiting toxicity, up to three additional patients will be enrolled at the same dose level.
Only in case of dose-limiting toxicity observed in two or more patients in the first cohort, further recruitment will be at the dose of 200 mg qd for sorafenib (cohort –1).

The maximum tolerated dose will be defined when two or more patients in a cohort of six patients experience a dose-limiting toxicity. The recommended dose (RD) for the second part of the study will be the dose level immediately preceding, or, in the absence of a documented maximum tolerated dose, the dose of 400 mg bid.

9.2 Phase II

In the second part of the trial, the cohort of patients treated at the RD will be expanded, up to a total of 27 patients treated with vinorelbine 30 mg per square meter (days 1, 8 q21) plus sorafenib at the RD.

Six patients in this expanded cohort will undergo sample collection for pharmacokinetic analysis of vinorelbine and sorafenib.

9.3 Vinorelbine administration

Vinorelbine will be diluted in D5W or NS to a final concentration of 0.5-2 mg/mL, and administered as a 6-10 min infusion, through free-flowing IV, followed by an IV flush with 200 to 300ml NS or D5W.

Premedication (such as with dexamethasone, anti-nausea, or analgesics) will be according to internal directives for each center for the first administration, and then adjusted to each patient's need.

9.4 Sorafenib administration

- Bayer will supply sorafenib tablets to the pharmacy in each clinic, that will ensure appropriate storage.
- Tablets will be dispensed only to subjects enrolled in this clinical trial, as 200 mg capsules (Nexavar®).
- Drug intake will be approximately every 12 hours (or every 24 hours in case of dose decrease to level –1).
- Patients can take sorafenib fasting.
- Skipped doses will not be recuperated.
- Subjects will be asked to return all unused tablets at the end of treatment.

9.5 Duration of therapy

In both study phases, patients will receive up to a total of 8 cycles of combination therapy with vinorelbine and sorafenib, unless disease progression or unacceptable toxicity is manifested, or a patient withdraws informed consent.
In case of late response, patients can receive more than 8 cycles of combination therapy, up to 4 cycles after achievement of the best response obtained.

After completion of the combination therapy, those patients whose disease has not progressed during the combination treatment will continue receiving sorafenib at the dose of 400 mg bid alone. For patient treated in the combination therapy with a dose of sorafenib lower than 400 mg bid, dose escalation to sorafenib 400 mg bid will be made 2 weeks after the last infusion of vinorelbine, if criteria for retreatment are met.

10. PATIENT ASSESSMENT

10.1 Screening procedures

All patients will have blood tests and urinalysis within two weeks from therapy start.

Within 4 weeks prior to therapy start, patients will have appropriate radiological imaging to document baseline disease, according to RECIST criteria, for targeted lesions (see Appendix E), including a bone scan and a baseline chest x-ray (unless CT or MRI scans of the thorax are performed) and an abdomen ultrasound (unless CT or MRI scans of the abdomen are performed). Brain CT or MRI scans are only required in the presence of

symptoms suggestive for CNS involvement.

Within 4 weeks prior to therapy start, patients will have appropriate cardiac evaluation, by either multigated acquisition (MUGA) scan or echocardiogram (ECHO) and 12-lead ECG.

Past and present medical/oncological history including concomitant medications, physical examination, signs and symptoms and ECOG performance status will be assessed within two weeks from therapy start.

Patients with difficult peripheral venous access should be proposed to have the placement of a central access device, followed by complete wound healing before therapy start.

Once the eligibility of a patient is confirmed, the informed consent will be signed both by the investigator proposing the participation to the trial, and by the patient.

10.2 Monitoring of patients treated with combination therapy

During administration of sorafenib and vinorelbine, in both study phases, patients will be routinely assessed for adverse events and cell blood count on days 1 and 8 of every cycle.

A complete biochemical blood analysis will be routinely performed at day 1 of each cycle.

Blood samples will be collected on the same day of therapy administration, or, in case of necessity, within the preceding 24 hours.

Physical examination, laboratory testing and evaluation of adverse event will be performed more often, if clinically indicated.

Only for patients treated during the phase I (dose escalation) part of the study, additional blood samples will be collected for cell blood count on day 15 of the first two cycles and for hepatic function markers on day 8 and 15 of the first two cycles.
The utility of maintaining these additional tests for patients treated in the phase II cohort will be discussed by the investigators after completion of the phase I.

Radiological imaging will be repeated regularly every 6 weeks (two cycles), unless all measurable disease is assessable by physical exam.
Assessment of tumor response will be performed as long as the patient remains on study, or until disease progression for patients removed from the study for other reasons.
Patients who achieve complete or partial response will be re-evaluated at least 4 weeks later to confirm the initial observation of response.

A cardiac re-evaluation through 12-lead EKG plus echocardiogram or MUGA scan will be repeated every 12 weeks (4 cycles), or more often if clinically indicated.

10.3 Monitoring of patients entering monotherapy with sorafenib

Patients receiving sorafenib monotherapy, after documented disease control, will be assessed for adverse events, cell blood count and biochemical blood analysis on day 1 of 21-day cycles for the first 8 cycles of sorafenib monotherapy, and every 2 cycles (42 days) thereafter, until patient's removal from the study.
Physical examination, laboratory testing and evaluation of adverse events will be performed more often, if clinically indicated.

Radiological imaging will be repeated regularly every 6 weeks (two cycles), unless all measurable disease is assessable by physical exam, as long as the patient remains on study, or until disease progression for patients removed from the study for other reasons.

A cardiac re-evaluation, through 12-lead EKG plus echocardiogram or MUGA scan will be repeated every 12 weeks (4 cycles), or more often if clinically indicated.

Chest X-ray, bone scan, abdomen ultrasound will be repeated if clinically indicated or, for patients in study more then 12 months, approximately once a year.

All patients will be followed for a minimum of two years for survival.

Table 2.
Schematic layout of procedures planned for regular assessment of patients on study.

	**Screening**		**Combination treatment**			**Monotherapy**	
						**≤ 168 days**	**> 168 days**
	≤ 28 days	≤ 14 days	D. 1	D. 8	D. 15	Every 21 days	Every 42 days
CT-MRI scans of target lesions	X		X[1]			X[1]	X
Chest X-ray[2,3]	X						
Bone scan[3]	X						
Abdomen ultrasound[3]	X						
12-lead ECG[3,4]	X		X[4]			X[4]	X[4]
2-D ECHO or MUGA[3,4]	X		X[4]			X[4]	X[4]
Brain CT-MRI scan[5]	(X)						
Medical/oncological history		X	X	X		X	X

Physical examination		X	X	X		X	X
Signs/symptoms		X	X	X		X	X
Concomitant medications		X	X	X		X	X
ECOG PS		X	X	X		X	X
Adverse events			X	X		X	X
Drug compliance			X	X		X	X
Hematology[6]		X	X	X	X[7]	X	X
Blood chemistry[8]		X	X	X[7,9]	X[7,9]	X	X
Coagulation[10]		X					
Urinalysis[11]		X					

[1] To be repeated every 2 cycles (unless all target lesions clinically measurable), before day 1 of the following cycle.

[2] Only if CT-MRI scan of the thorax not planned.

[3] To be repeated if clinically indicated, otherwise approximately once a year.

[4] To be repeated every 4 cycles (~84 days), or more often if clinically indicated

[5]Only if clinically indicated.

[6] Comprehensive of: hemoglobin (Hgb), platelet count (Plt), white blood cell count (WBC), white blood cell differential.

[7] Only for the first 2 cycles in patients treated on the phase I part. If retained necessary, also in patients treated at the RD.

[8] Comprehensive of: total bilirubin, alanine transaminase (ALT), aspartate transaminase (AST), alkaline phosphatase, total protein, albumin, sodium, potassium, chloride, calcium, blood urea nitrogen (BUN)/urea, creatinine, lipase, amylase, glucose.

[9] Only: alanine transaminase (ALT), aspartate transaminase (AST)

[10] Comprehensive of: prothrombin time (PT), international normalized ratio (INR), partial thromboplastin time (PTT)

[11] Comprehensive of: pH, ketones, protein, glucose, WBC/HPF, RBC/HPF

11. TOXICITY AND DOSE DELAYS - MODIFICATIONS

11.1 Criteria for retreatment

If disease progression is not observed, patients will be retreated at the same dose level of cycle 1, unless a dose reduction is necessary as a consequence of toxicity, according to criteria listed below.

Hematological toxicity is expected to occur mainly as a consequence of treatment with vinorelbine.

In order to receive vinorelbine therapy, both on day 1 and 8 of each cycle, patients must have ANC $\geq 1.0 \times 10^9/L$ and platelets $\geq 75 \times 10^9/L$.

No sorafenib interruption is mandatory in case of hematological toxicity.

In case of diarrhea, skin rash, hand-foot syndrome or hypertension, most probably attributable to sorafenib intake, the administration of vinorelbine will be allowed, at investigator's discretion.

11.2 Hematological toxicity

In case of neutropenia ≥G3 (ANC <1.0 x 10^9/L) or thrombocytopenia ≥G2 (platelet count <75 x 10^9/L) on day 1 of a cycle, chemotherapy will be withheld until resolution to ANC ≥1.0 x 10^9/L and platelets ≥75 x 10^9/L, with no need for dose-modification.

In the case of neutropenia ≥G3 or thrombocytopenia ≥G2 on day 8, the second dose of vinorelbine will be cancelled, and the treatment will be restored on day 1 of the next cycle.

If in a patient vinorelbine is postponed on day 1, or cancelled on day 8 two times as a consequence of neutropenia, the use of prophylactic G-CSF will be allowed from the following cycle. Colony-stimulating factors will be started 24-48 hours after the vinorelbine dose, and discontinued at least 24 hours prior to the next administration.

The use of stimulators of thrombopoiesis is not allowed.

If in the same patient vinorelbine infusion is postponed on day 1, or cancelled on day 8 twice as a consequence of thrombocytopenia, a dose reduction of vinorelbine is allowed (see Section 12.8).

No dose modification/reduction is mandatory for vinorelbine in case of anemia G1-2. If the anemia results in symptoms (such as fatigue) or G3 (<80 g/L), then this can be treated appropriately, with erythropoietin and/or blood transfusions.

11.3 Diarrhea

The appearance of diarrhea is expected as a consequence of sorafenib administration.

In the case of diarrhea, loperamide intake, according to manufacturer's instructions, should be initiated after the third episode on the same day.

For diarrhea Grade ≤2 (increase of up to 6 stools per day over baseline), sorafenib suspension is not mandatory, but appropriate therapy with loperamide is mandatory in the presence of diarrhea Grade 2.

If Grade 2 diarrhea persists for more than 5-7 days despite loperamide, a temporary suspension of sorafenib until toxicity resolution to Grade 1 should be considered.

Sorafenib temporary suspension is mandatory in the case of diarrhea G3-4, until

resolution to Grade ≤2.

If diarrhea persists as G3-4 longer than 24 hours after sorafenib suspension and starting adequate therapy with loperamide or diphenoxylate/atropine, sorafenib will be eventually restarted at a lower dose level (see Section 12.8).

11.4 Hand-foot skin syndrome (HFS)

As a consequence of treatment with sorafenib, patients may experience a reddening and soreness of the skin, particularly on the surfaces of the hand and feet but also in skin folds. This toxicity is referred to as hand-foot syndrome (HFS).

HFS will be graded as following:

- Grade 1: painless mild erythema, swelling, or desquamation nor interfering with daily activities;
- Grade 2: painful erythema, desquamation, or swelling interfering with, but not precluding normal physical activities; or small blisters or ulcerations less than 2 cm in diameter;
- Grade 3: blistering, ulcerations, swelling or severe pain interfering with walking or performing normal daily activities; cannot wear regular clothing;
- Grade 4: diffuse or local process causing serious infectious complications, or a bedridden state or hospitalization.

Patients will be provided recommendations to decrease its incidence and severity.

In the presence of Grade ≥2 HFS, patients must be aware of the need to hold sorafenib intake, until resolution to Grade ≤1 HFS.

A dose level decrease (see Section 12.8) will be applied for patients presenting Grade 3-4 HFS, or two separate episodes of Grade 2 HFS with an interval shorter than 3 months.

11.5 Skin rash

Sorafenib treatment will continue in the presence of rash G1 and, at investigator's discretion; G2, and the use of topical products, such as emollients or low potency topical steroids, will be allowed.

In case of rash of Grade 3, patients will hold sorafenib administration, until resolution to Grade 1.
If Grade3 rash persists more than 5 days after suspension of sorafenib, or if a second Grade3 rash of any duration occurs within less than 3 months, then the treatment will be restarted at one dose reduction (see Section 12.8).

11.6 Hypertension

Hypertension will be graded as G1 if transient and asymptomatic, not requiring treatment.

If a patient develops recurrent or persistent or symptomatic hypertension (G2), she will receive appropriate anti-hypertensive treatment, starting with a monotherapy.

Only in case of diastolic blood pressure >100 mm/Hg or symptomatic G2 hypertension, sorafenib will be suspended until diastolic blood pressure decreases to <100 mm/Hg, and symptoms resolve.

In case of G3 hypertension, requiring more than one drug, sorafenib treatment will be suspended until achievement of a good blood pressure control, and eventually resumed at a lower dose (see Section 12.8).

In case of hypertension G4 (e.g. hypertensive crisis), treatment with sorafenib will be permanently discontinued.

11.7 Cardiac toxicity

A Grade 3-4 cardiac event is defined as:

- a LVEF <40%, or
- a 20% decline or greater in LVEF from baseline to <50%, or
- any decrease to a value below the lower limit of normal in LVEF that is symptomatic at rest for dyspnea, orthopnea, S3 gallop, tachycardia or inspiratory crackles.

In the presence of a grade ≥3 cardiac event, sorafenib will be permanently discontinued, and the patient will be considered off-study.
The responsible investigator will evaluate the utility of maintaining a vinorelbine-containing treatment.

For Grade2 asymptomatic events, with a decrease in LVEF to 40-50%, sorafenib administration will be temporary withheld.
The assumption of sorafenib will be allowed if within 4 weeks from its suspension LVEF recovers above 50%. If sorafenib treatment is restored, LVEF will be assessed approximately every 28 days for the next 2 cycles, then every 2 cycles.

In case of a second LVEF drop to 40-50%, sorafenib assumption will be permanently discontinued.

11.8 Other non-hematological toxicities

Other toxicities will be evaluated as possibly or likely related to each study drug (also see

Section 15.2).

In the presence of adverse events that are unlikely related to the study treatment, the investigator will evaluate whether a temporary suspension of one or both drugs is necessary.

Any possibly study-related Grade ≥3 adverse event (with the exception of nausea or vomiting that has not been adequately premedicated, and vinorelbine-induced peripheral phlebitis or extravasation ulceration) will result in the suspension of the possibly responsible drug(s), until resolution to Grade 1, with a subsequent dose reduction in the following cycles.

In case of Grade 2 toxicities possibly related to study treatment, no dose suspension/reduction is mandatory, but the investigator will evaluate the need of modifying treatment schedule in case of recurrent toxicities.

11.9 Dose modifications

No intra-patient dose escalation for sorafenib will be allowed during the combination with vinorelbine.

Any patient experiencing at any cycle a toxicity requiring a dose reduction, will be evaluated by the investigator, who will evaluate the risks for the patient to restore therapy and the utility of continuing the study treatment at lower doses, rather than definitively terminating her participation in this trial. The investigator must be aware of the multiple therapeutic possibilities that are usually available for patients at this stage of disease.

Patients experiencing vinorelbine-induced toxicities requiring a dose reduction will be tretated at a dose of 25 mg/m^2, and then 20 mg/m^2, if a second dose reduction is necessary. No further dose reductions will be allowed.

Sorafenib dose reduction is mandatory for patients experiencing a DLT (see Section 12), and recommended for patients experiencing the need of frequent drug suspensions (>twice in 2 months).
Sorafenib dose can be lowered from 400 mg bid to 200 mg bid, and from 200 mg bid to 200 mg qd (up to a maximum of two dose-reductions for patients treated in cohort 2 dose level).

If a patient undergoes sorafenib dose reduction(s) during combined treatment with vinorelbine, the same dose will be maintained as monotherapy treatment, in the absence of progressive disease after completion of chemotherapy.

12. DEFINITION OF DOSE-LIMITING TOXICITY (DLT)

Dose-limiting toxicity is defined as one or more of the following:

1) Febrile neutropenia, defined as a temperature ≥38.5°C in the presence of ANC $<1.0x10^9/L$.
2) Grade4 neutropenia lasting more than 5 days.
3) Grade4 thrombocytopenia ($<25 \times 10^9/L$) or anemia (<65 g/L).
4) Grade3 thrombocytopenia ($<50 \times 10^9/L$), if lasting more than 5 days.
5) Grade3AST-ALT increase (>5.0 x ULN), if lasting more than 5 days.
6) Grade3 rash, if lasting more than 5 days after suspension of sorafenib.
7) Grade3 diarrhea, if lasting more than 24 hours from suspension of sorafenib and adequate treatment with loperamide or diphenoxylate/atropine.
8) Other non-hematological toxicity of Grade≥3, except unpremedicated nausea or vomiting, vinorelbine-induced peripheral phlebitis or extravasation ulceration.
9) Impossibility to start the second cycle of therapy within 14 days from the planned day, as a consequence of toxicity.

Any patient experiencing a DLT at any cycle will be evaluated by the investigator, who will assess the utility for the patient of continuing the study treatment at lower doses, rather than definitively terminating his participation to this trial.

No patient experiencing a DLT will be retreated at the same dose. The investigator will evaluate risks and benefits for the patient, being aware of the multiple therapeutic possibilities that are usually available for patients at this stage of disease.

13. CONCOMITANT MEDICATIONS

13.1 Anticancer treatments

No other chemotherapy, anticancer hormonal therapy, or experimental anticancer medications will be permitted during the treatment period.

Biphosphonates for known bone metastases will be allowed only if started prior to or at the same time trial therapy is initiated, but will not be allowed to be initiated during the study participation.

Patient requiring radiation therapy during the study treatment will remain on study only if the metastases do not represent progression of disease.
In these cases, patients should have study drugs withheld during the course of radiation therapy and until recovery (at least 8 days after radiation).
If there is no evidence of radiation recall reaction, such patients may be retreated with study drugs, provided that there is no sign of progression of disease.

13.2 Surgical procedures

In the event surgery is necessary during trial participation, both sorafenib and vinorelbine should be stopped two weeks before surgery, and resumed after complete wound healing, if there is no sign of disease progression.

13.3 Allowed medications

Patients will continue therapies initiated before the study start for concomitant diseases, and will receive optimal treatment for adverse events or newly diagnosed pathologies during the participation to the study. Nevertheless, any disease progression requiring other forms of anti-tumor therapy will result in discontinuation of the subject from the trial.

In particular, the following will be permitted during the trial:

- antiemetics;
- erythropoietic agents;
- skin topical products, such as emollients, low potency topical steroids or topical antibiotics;
- loperamide, or similar antidiarrhoics;
- anti-inflammatory or narcotic analgesics;
- antihypertensive medications.

All concomitant medications will be recorded with indication, dose information and dates of administration.

13.4 Hematopoietic growth factors

Prophylactic use of hematopioetic growth factors will be permitted for specific patients, only after documented difficulty to maintain an adequate dose-intensity of vinorelbine as a consequence of recurrent neutropenia.

If in a patient vinorelbine is postponed on day 1 three times, or cancelled on day 8 two times as a consequence of neutropenia, the use of profilactic G-CSF will be allowed in the following cycles. Colony-stimulating factors can be started 24 hours after a vinorelbine dose, and discontinued at least 24 hours prior to the next administration.

Use of stimulators of thrombopoiesis will not be allowed.
Packed red blood cell and platelet transfusions should be administered as clinically indicated.

14. DISCONTINUATION OF THERAPY AND WITHDRAWAL FROM STUDY

A patient will stop receiving the study treatment and will be withdrawn from the study when any of the criteria is met:

- Progression of disease based on physical examination or radiological

measurements.

- Patient's inability to tolerate therapy despite dose reduction. This includes allergic reactions not controlled with slower infusions and routine pre-medications.
- Patient experiencing an adverse event that would mandate a dose reduction below 200 mg qd for sorafenib, or below 20 mg/m^2 for vinorelbine.
- Withdrawal of informed consent.
- Patient evaluated as non-compliant by the investigator.
- Need of new anticancer treatments. Patient requiring palliative radiation therapy should have study drugs withheld during the course of radiation therapy and until recovery (at least 15 days after radiation). Such patients may be retreated with study drugs, provided that the metastases do not represent progression of disease and there is no evidence of radiation recall reaction.
- Patient with clinical signs or laboratory test results consistent with pregnancy. The pregnancy will be followed up via the Pregnancy Monitoring Form.

The investigator can discontinue the patient at any time, at his/her discretion.

15 ASSESSMENT AND REPORTING OF ADVERSE EVENTS

15.1 Definition of Adverse Event (AE)

An adverse event (AE) is any untoward medical occurrence in a patient or clinical investigation subject administered a pharmaceutical product/biologic (at any dose), or medical device and which does not necessarily have to have a causal relationship with this treatment.

This includes the onset of new illness and the exacerbation of pre-existing conditions, whether or not considered related to the medicinal product.

An AE can therefore be any unfavorable and unintended sign (including an abnormal laboratory finding), symptom or disease temporally associated with the use of the study treatment, as:

- occurring in the course of the use of the study drugs;
- associated with, or observed in conjunction with product abuse or overdose, whether accidental or intentional;
- associated with, or observed in conjunction with product withdrawal.

All adverse events will be recorded in the patient's medical records and on the data collection documents. The onset and end date, initial severity, change in severity grade, casual relationship to the study treatment will be recorded for each adverse event.

The severity of the adverse event and relationship to the study drug will be assessed according to specific guidelines described below.

15.2 Assessment of adverse events severity and relationship to treatment

For toxicity evaluation and grading of each AE, all the investigators will refer to the NCI Common Toxicity Criteria Version 3.0, available on-line (Appendix D).

For adverse events not covered by the common toxicity criteria, the following definitions will be used:

- Mild (G1): awareness of sign, symptom or event, but easily tolerated.
- Moderate (G2): discomfort enough to cause interference with usual activity and may warrant intervention.
- Severe (G3): incapacitating with inability to do usual activities or significantly affects clinical status, and warrants intervention.
- Life-threatening (G4): immediate risk of death.
- Causing death (G5).

The investigator, based on available information, will assess the relationship of any AE to the use of sorafenib and/or vinorelbine. A causal role of each drug will be defined according to the following guidelines:

- Likely: temporal association, other etiologies unidentified and unlikely.
- Possible: temporal association, but other etiologies are likely the cause; however, involvement of the drug cannot be excluded.
- Unlikely: no temporal association, or the cause of the event has been identified, or the drug cannot be implicated.

All AEs, including abnormal laboratory values, will be recorded on the adverse event module.

15.3 Monitoring adverse events

Patients reporting an AE will be monitored with appropriate clinical assessments and laboratory tests, as determined by the treating physician.

All adverse events must be followed to satisfactory resolution or stabilization, also after patient's withdrawal from the study.

Any measures taken and follow-up results will be recorded on the data collection form, as well as in the patient's source document. Follow-up laboratory results should be filed with the patient's source documentation.

15.4 Definition of Serious Adverse Event (SAE)

A serious adverse event (SAE) is any adverse event that results in any of the following

outcomes:

- death;
- life-threatening AE (that places the subject, in the view of the initial reporter, at immediate risk of death from the AE as it occurs, i.e., suicidal ideation/suicide attempt);
- persistent or significant disability/incapacity;
- required in-patient hospitalization, or prolonged hospitalization, with exceptions listed below;
- congenital anomaly or birth defect.

Additionally, important medical events that may not result in death, be life-threatening, or require hospitalization may be considered a SAE when, based upon appropriate medical judgment, they may jeopardize the subject and may require medical or surgical intervention to prevent one of the outcomes listed in this definition.
Examples of such medical events include allergic bronchospasm requiring intensive treatment in an emergency room or at home, blood dyscrasias, or convulsions that do not result in in-patient hospitalization, or the development of drug dependency or drug abuse.

"Serious" and "severe" as stated on the AE collection form are not synonymous. Severity refers to the intensity of a reaction (i.e., mild, moderate, severe, etc.). "Serious" refers to a regulatory definition for the outcome of an event (i.e., fatal, life-threatening, resulted in hospitalization, etc.).

If a subject has a preplanned hospitalization or procedure (e.g., elective surgery) during the study for an event, which occurred before the study start, the hospitalization is considered a therapeutic intervention and not an SAE. However, the event and/or the procedure must be reported in the CRF.

An AE requiring/prolonging in-patient hospitalization will not be considered a SAE for this study if the only reason for the hospitalization or prolongation is one of the following ones:

- administration of chemotherapy;
- transfusion of blood products;
- administration of study procedure;
- placement of a permanent intravenous catheter;
- hospice placement for terminal care

15.5 Report of Serious Adverse Events (SAEs)

Reports of all SAEs, including deaths must be communicated to the appropriate

Institutional Review Board or ethical review committee and/or reported in accordance with local law and regulations.

Any serious adverse event (SAE), whether or not deemed drug-related or expected, must be reported by the physician immediately or within 24 hours to:

McGill University, Department of Oncology
c/o Penny Chipman, Manager Clinical Research Program
546 Pine Avenue West, Montreal, Quebec; H2W 1S6
Fax: 514-398-2754

Bayer Inc.
Bayer Healthcare Pharmaceuticals
Drug Safety
77 Belfield Road, Toronto, Ontario
M9W 1G6
Tel: (416) 240-5230 / 1-800-622-2937 ext. 5230
Fax: (416) 614-0516 / 1-866-232-0565

Questions to:
DSI Canada@bayer.com

If the report is made via telephone, a written report must follow as soon as possible, which includes a full description of the event and any sequelae. SAEs that occur at any time after the inclusion of the subject in the study (defined as the time when the subject signs the informed consent) up to 30 days after the subject completed or discontinued the study must be reported.

The subject is considered to have completed the study either after the completion of the last visit or contact (eg, phone contact with the investigator), or after the last dose of study medication, whichever is later.

The date of discontinuation is when a subject and/or investigator determines that the subject can no longer comply with the requirements for any further study visits or evaluations (e.g. the subject is prematurely discontinued from the study).

15.6 Reporting of a subject's death

The death of any subject during the study or within the 30 days post-treatment period following study completion (as defined above), regardless of the cause must be reported as a SAE within 24 hours to:

McGill University, Department of Oncology
c/o Penny Chipman, Manager Clinical Research Program

546 Pine Avenue West, Montreal, Quebec H2W 1S6
Fax: 514-398-2754

Bayer Inc.
Bayer Healthcare Pharmaceuticals
Drug Safety
77 Belfield Road, Toronto, Ontario
M9W 1G6
Tel: (416) 240-5230 / 1-800-622-2937 ext. 5230
Fax: (416) 614-0516 / 1-866-232-0565

Questions to:
DSI Canada@bayer.com

If the report is made via telephone, a full written report (SAE form and death page from the CRF) must follow as soon as possible. The death must be recorded in the patient chart and on the CRF. If an autopsy is performed, the report must be provided to McGill University, Department of Oncology and Bayer Canada.

After 30 days from study completion, a patient's death will be reported for regular follow-up purposes, but will not be reported as a SAE, unless considered by the investigator possibly related to a previous study-related AE.

15.7 Report of pregnancies

Although not considered a SAE (unless an event occurs with a serious outcome), if a subject should become pregnant during the course of the study, it must be reported within 24 hours to:

Bayer Inc.
Bayer Healthcare Pharmaceuticals
Drug Safety
77 Belfield Road, Toronto, Ontario
M9W 1G6
Tel: (416) 240-5230 / 1-800-622-2937 ext. 5230
Fax: (416) 614-0516 / 1-866-232-0565

Questions to:
DSI Canada@bayer.com

Bayer representatives will provide instructions on how to collect pregnancy information.

If a pregnancy occurs in a subject, study drugs should be discontinued. Follow-up information on the outcome of the pregnancy should also be forwarded to the sponsor.

16. STATISTICAL CONSIDERATIONS

16.1 Sample size justification for Phase II

A pooled analysis of data available in the literature was performed, selecting for phase II trials with vinorelbine administered as monotherapy as first-line treatment for metastatic breast cancer. From this analysis, an overall response rate of 43.2% is estimated as representative of vinorelbine single-dose activity in this type of patients (188 objective responses out of 435 evaluable patients).

The combination with sorafenib, increasing vinorelbine efficacy, is reasonably expected to improve the response rate compared to vinorelbine monotherapy.
Even though the attainment of an objective response does not directly correlate with a real survival advantage for patients, the higher is the advantage in overall response rate for the combination therapy, the more suggestive this would be of a clinical promising synergism.

It is felt that if the two drugs combination would lead to an increase from ~43% to 63% of response rate (+ 20%), this would compel us to further investigate the activity in a randomized trial.

Sample size for the Phase II was determined using simulation, to obtain a power of 80% in order to detect such an increase in response rate with a significance level (alpha) of 0.10.
Based on this assumption, the minimum sample size would be 27 evaluable patients, as shown below:

QuickTime™ and a
TIFF (LZW) decompressor
are needed to see this picture.

A binomial test with a nominal 0.10 one-sided significance level will have the power as shown below for various response rates to test the Null hypothesis response rate of 43%, with a sample size of 27 patients.

Null hypothesis response rate (p0)	Alternative hypothesis response rate (p1)	Power
43%	53%	40%
43%	58%	61%
43%	63%	80%
43%	68%	92%

Standard software package nQuery Advisor was used to calculate the sample size required for this trial.

16.2 Definition of study outcomes

Objective response (OR)	Confirmed complete response (CR) or partial response (PR), defined according to RECIST criteria, persisting ≥4 weeks after the initial documentation.
Clinical benefit (CB)	Confirmed objective responses plus confirmed stable disease (SD ≥ 24 weeks), according to RECIST criteria.
Time to response (TTR)	Time from the first dose of study medication to the first documentation of an objective tumor response.
Duration of response (DR)	Time from first objective status assessment of CR or PR to the first time of progression or death as a result of any cause.
Time to progression (TTP)	Time from the first dose of study medication to the first documentation of an objective tumor progression.<br>TTP will be censored at the date of death for patients who have not had objective disease progression.
Progression-free survival (PFS)	Time from the first dose of study medication to the first documentation of an objective tumor progression, or to death on-study, whichever occurs first.
Overall survival (OS)	Time from study enrollment to time of death as a result of any cause.<br>Survival time will be censored at the date of the last follow-up visit for patients who are still alive.

16.3 Analysis populations

- Intent-to-Treat (ITT) Population: all subjects who are enrolled in the trial.
- Safety Population: all subjects who receive at least 1 dose of trial medication(s).

All efficacy analyses, except for analyses on OR and DR, will be performed on the ITT population.

Analyses on OR will be performed for subjects who receive at least 1 dose of trial medication(s).

Analyses of DR will be performed for overall responders only.

Safety population will be the primary population for evaluating treatment administration/compliance and safety.

16.4 Analysis of primary endpoints

Confirmed objective responses and stable diseases will be calculated, to determine objective response rate (ORR) and clinical benefit rate (CBR). The 95% confidence interval for ORR and CBR will be constructed using the exact methods.

ORR is defined as the proportion of subjects with confirmed CR or confirmed PR according to the RECIST criteria, relative to the total subjects who receive at least 1 dose of study medication(s).

CBR is defined as the proportion of subjects with clinical benefit response, relative to the total subjects who receive at least 1 dose of study medication(s).

Patients who do not have on-study radiographic tumor re-evaluation (or clinical re-evaluation, when physical examination is indicated to monitor disease state) will be counted as non-responders/non-benefiters in the assessment of ORR and CBR, unless replaced through enrollment of exta-evaluable patients.

16.5 Analysis of event-free times

The efficacy analysis will include Kaplan-Meier curves for TTP, PFS and OS, including quartiles for each variable. The 80% confidence interval of the median event-free time will be estimated using the standard error derived by Greenwood's formula. Covariates will not be included in the calculation of median survival time.

Kaplan-Meier analysis (Kaplan and Meier 1958) will be done using PROC LIFETEST in Statistical Application Software (SAS) (SAS Institute, Inc. 1989), or other equivalent statistical package. Other analyses will be done as necessary.

Kaplan-Meier curves and quartiles will illustrate also time to response and duration of response, if a sufficient number of responders is observed.

16.6 Safety analysis

The safety analysis will include descriptive summary statistics and listings of adverse events, CTCAE, laboratory data, vital signs, growth factor usage, and transfusions.

The AE reporting experience begins from the time that the subject receives the first study drug, and ends 28 days after the last dose of study drugs(s) is administered.

Detailed information collected for each AE will include a description of the event, duration, whether the AE was serious, intensity, estimated relationship to study drug(s), action taken, clinical outcome.

17. PHARMACOKINETICS (PK) ANALYSIS

Once the recommended dose is defined, pharmacokinetics (PK) analysis will be performed in a subset of six patients treated at this dose.
In these patients, the administration of sorafenib at the first cycle will start at day 4.

The analysis will compare PK of each drug in the presence or absence of significant concentrations of the other one.

Bayer will analyze the collected samples for both sorafenib and vinorelbine concentration, and will provide the obtained results to the Principal Investigator.

For collection, processing and shipping of PK samples, please see Appendix F.

17.1 PK analysis of vinorelbine

Plasma samples for vinorelbine will be collected on day 1 of the first cycle (before sorafenib treatment, which will begin on day 4 of cycle 1), and day 1 of the second cycle, after attaining a steady state in sorafenib plasma levels.

In both cases, collection time points for the samples will be: time 0 (pre-dose), at the end of the infusion, and then at 0.5, 1, 2.5, 5, 7, 24, 48, 72 hours from the end of the infusion. AUC, C_{max}, C_{min} and $t_{1/2}$ will be determined, comparing before and after sorafenib intake.

17.2 PK analysis of sorafenib

Sorafenib PK will be analyzed after attainment of steady state levels, comparing before and after an administration of vinorelbine.
Plasma samples for sorafenib PK will be collected on day 21 of the first cycle (before vinorelbine administration planned for the following day) and on the following day 1 of the second cycle, after vinorelbine administration.

For both days, collection time points will be: time 0, 0.5, 1, 2.5, 5, 7, 24 hours after the

first daily intake, plus 48 and 72 hours after the first sorafenib intake on cycle 2 day 1. AUC, C_{max}, and C_{min} will be determined, comparing before and after vinorelbine administration

Table 3
Planned time points for collection of PK samples, with relative planned treatment.

DAY	TIME (hours)	Vinorelbine INFUSION	Sorafenib INTAKE	Vinorelbine PK SAMPLE	Sorafenib PK SAMPLE
Cycle 1 day 1	0			X	
	0 → 0.1	X			
	0.1			X	
	0.5			X	
	1			X	
	2.5			X	
	5			X	
	7			X	
Cycle 1 day 2	0 (24)			X	
Cycle 1 day 3	0 (48)			X	
Cycle 1 day 4	0 (72)		X	X	
	12		X		
Cycle 1 day 8	0 → 0.1	X	X		
	12		X		
Cycle 1 day 21	0		X		X
	0.5				X
	1				X
	2.5				X
	5				X
	7				X
	12		X		
Cycle 2 day 1	0		X	X	X
	0 → 0.1	X			
	0.1			X	
	0.5			X	X
	1			X	X
	2.5			X	X
	5			X	X
	7			X	X
	12		X		
Cycle 2 day 2	0 (24)		X	X	X
	12 (36)		X		
Cycle 2 day 3	0 (48)		X	X	X
	12 (60)		X		
Cycle 2 day 4	0 (72)		X	X	X
	12 (84)		X		

18. DATA HANDLING AND RECORD KEEPING

18.1 Case Report Forms (CRFs)

Specific Case Report Forms (CRFs) will be created by the principal investigator, either as paper forms or as electronic data record, and distributed to all participating centers.

CRFs will be completed for each patient enrolled in the study by the Clinical Research Associate responsible in each center overseen by the Principal Investigator.
For each completed CRF, a copy will remain at the treating center, and a second identical copy will be sent for collection to the principal investigator, at the coordinating center (Jewsih General Hospital, Montreal), for definitive analyses.

The completed original CRFs will be property of McGill Unversity.

It is each investigator's responsibility to ensure accurate completion and review of all the necessary CRFs, that will be signed by the compiling investigator or by an authorized staff member. These signatures will attest that the information contained on the CRFs is true.

18.2 Record retention

To enable evaluations and/or audits from regulatory authorities or Bayer, the investigator responsible in each center will keep records, including the identity of all participating subjects (sufficient information to link records, e.g., CRFs and hospital records), all original signed informed consent forms, copies of all CRFs, serious adverse event forms, source documents, and detailed records of treatment disposition.

The records will be retained by the investigator for 25 years.

If an investigator relocates, retires, or for any reason withdraws from the trial, the trial records will be transferred to an acceptable designee, such as another investigator, or another institution.

19. ETHICS

19.1 Institutional Review Board (IRB)/Independent Ethics Committee (IEC)

It is the responsibility of the investigator in each participating center to have prospective approval of the trial protocol, protocol amendments, informed consent forms, and other relevant documents from the IRB/IEC.

The investigator will also retain all correspondence with the IRB/IEC.

Copies of the IRB/IEC approvals will be forwarded to McGill Department of Oncology and to Bayer.

The only circumstance in which an amendment may be initiated prior to IRB/IEC approval is where the change is necessary to eliminate apparent immediate hazards to the subjects. In that event, the investigator must notify the principal investigator at the coordinating center and the IRB/IEC in writing within 5 working days after the implementation. The principal investigator will subsequently notify Bayer.

19.2 Ethical conduct of the trial

The trial will be performed in accordance with the protocol, local regulatory requirements and laws and recommendations guiding physicians in biomedical research involving human subjects, as reported in the Declaration of Helsinki (Appendix A).

19.3 Subject information and consent

All parties will ensure protection of subject personal data and will not include subjects names on any sponsor forms, reports, publications, or in any other disclosures. In case of data transfer, Bayer will maintain high standard of confidentiality and protection of subject personal data.

The informed consent form, or any change made during the course of the trial, must be prospectively approved by the IRB/IEC of each participating center.

The responsible investigator in each center must ensure that each trial subject, or her legally acceptable representative, is fully informed about the nature and objectives of the trial and possible risks associated with participation.

The investigator, or a person designated by the investigator, will obtain written informed consent from each subject or the subject's legally acceptable representative before any trial-specific activity is performed (screening evaluations are not considered as trial-specific). The investigator will retain the original of each subject's signed consent form.

20. PUBLICATION POLICY

The results of this trial will be published regardless of the results. The first author will generally be the chair of the study. Additional authors, up to a maximum of 15, will be those who have made the most significant contribution to the overall success of the study.

The contribution will be assessed, in part but not entirely, in terms of patients enrolled and will be reviewed at the end of the trial by the study chair.

It will be the responsibility of the study chair to write up the results of the study within a reasonable time of its completion.

Material may not be submitted for presentation or publication without prior review by sponsor, co-investigators, study coordinator, and approval of the study chair. Review of material by Bayer for an abstract or a publication must be within 60 days.

21. REFERENCES

1. Esteva FJ, Valero V, Pusztai L et al. Chemotherapy of metastatic breast cancer: what to expect in 2001 and beyond. *Oncologist*, 2001. 6:133-146.
2. Nabholtz JM, Senn HJ, Bezwoda WR et al. Prospective randomized trial of docetaxel versus mitomycin plus vinblastine in patients with metastatic breast cancer progressing despite previous anthracycline-containing chemotherapy. *J Clin Oncol*, 1999. 17:1412-1424.
3. Slamon DJ, Leyland-Jones B, Shak S et al. Use of chemotherapy plus a monoclonal antibody against HER2 for metastatic breast cancer that overexpresses HER2. *New Eng J Med*, 2001 344:783-792.
4. Mackey JR, Paterson A, Dirix LY et al. Final results of the phase III randomized trial comparing docetaxel (T), doxorubicin (A), and cyclophophamide (C) to FAC as first line chemotherapy (CT) for patients (pts) with metastatic breast cancer MBC). *Proc Am Soc Clin Onc*, 2002. Abstract 137.
5. Pierre Fabre Medicament. Internal report, 1993. PFM259 p.36(2).
6. Ashizawa T, Miyoshi K, Asada M et al. Anti-tumor activity of navelbine, a new vinca-alkaloid analog. *Gan To Kagaku Ryoho*, 1993. 20:59-66
7. Weisenberg RC. Microtubule formation in vitro in solutions containing low calcium concentrations. Science, 1972. 117:1104-1105.
8. Paintrand MR and Pignot I. Navelbine: an ultrastructural study of its effects. *J Electron Microsc*, 1983. 32:115-124.
9. Binet S, Fellous A, Lataste H et al. In situ analysis of the action of Navelbine on microtubules using immunofluorescence. *Semin Oncol*, 1989. 16(sup.4):5-8.
10. Wang LG, Liu XM, Kreis W, Budman DR. The effect of antimicrotubule agents on signal transduction pathways of apoptosis: a review. *Cancer Chemother Pharmacol*, 1999. 44:355-361.
11. Wang LG, Liu XM, Chao DL. Activation of MAP kinase during apoptosis mediated by vinorelbine in MCF-7 cells. *Proc Am Assoc Cancer Res*, 1999. 40:13.
12. Fumoleau P, Delgado FM, Delozier T et al. Phase II trial of weekly intravenous vinorelbine in first-line advanced breast cancer chemotherapy. *J Clin Oncol*, 1993. 11:1245-1252.
13. Canobbio L, Boccardo F, Pastorini G et al. Phase II study of Navelbine in advanced breast cancer. *Semin Oncol*, 1989. 16:33-36.
14. Weber BL, Vogel C, Jones S et al. Intravenous vinorelbine as first-line and second-line therapy in advanced breast cancer. *J Clin Oncol*, 1995. 13(11):2722-2730.
15. Terenziani M, Demicheli R, Brambilla C et al. Vinorelbine: an active, non cross-resistant drug in advanced breast cancer. Results from a phase II study. *Breast Cancer Res Treat*, 1996. 39(3):285-291.
16. Bruno S, Puerto VL, Mickiewicz E et al. Phase II trial of weekly i.v. vinorelbine as a single agent in first-line advanced breast cancer chemotherapy. The Latin-American experience. *Am J Clin Oncol*, 1995. 18(5):392-396.
17. Twelves CJ, Dobbs NA, Curnow A et al. A phase II, multicentre, UK study of vinorelbine in advanced breast cancer. *Br J Cancer*, 1994. 70(5):990-993.
18. Garcia-Conde J, Lluch A, Martin M et al. Phase II trial of weekly IV vinorelbine in first-line advanced breast cancer chemotherapy. Ann Oncol, 1994. 5(9):854-857; Romero A, Rabinovich MG, Vallejo CT et al. Vinorelbine as first-line chemotherapy for metastatic breast carcinoma. J Clin Oncol, 1994; 12(2):336-341).
19. Vogel C, O'Rourke M, Winer E et al. Vinorelbine as first-line chemotherapy for advanced breast cancer in women 60 years of age or older. *Ann Oncol*, 1999. 10:397-402.
20. Jones S, Winer E, Vogel C et al. Randomized comparison of vinorelbine and melphalan in anthracycline-refractory advanced breast cancer. *J Clin Oncol,* 1995.13(10);2567-2574.
21. Leung PP, tannock IF, Oza AM et al. Cost-utility analysis of chemotherapy using paclitaxel, docetaxel or vinorelbine for patients with anthracycline-resistant breast cancer. *J Clin Oncol*, 1999. 17(10):3082-3090.
22. Carmichael J, Hegg R, Firat D et al. Navelbine and fractionated dose doxorubicin improves first line advanced breast cancer. *Br J Cancer*, 1997. 77(sup.1):Abstr P85.
23. Ardavanis A, Mavroudis D, Kalbakis K et al. Pegylated liposomal doxorubicin in combination with vinorelbine as salvage treatment in pretreated patients with advanced breast cancer: a multicentre phase II study. *Cancer Chemother Pharmacol*, 2006. 58(6):742-748.
24. Namer M, Soler-Michel P, Turpin F et al. Results of a phase III prospective, randomised trial, comparing mitoxantrone and vinorelbine (MV) in combination with standard FAC/FEC in front-line therapy of

metastatic breast cancer. *Eur J Cancer*, 2001. 37(9):1132-1140.

25. Dieras V, Extra JM, Bellisant E et al. Efficacy and tolerance of vinorelbine and fluorouracil combination as first line chemotherapy of advanced breast cancer. *J Clin Oncol*, 1996. 14:3097-3104.
26. Ghosn M, Kattan J, Farhat F et al. Phase II trial of capecitabine and vinorelbine as first-line chemotherapy for metastatic breast cancer patients. *Anticancer Res*, 2006. 26(3B):2451-2456.
27. Savio G, Laudani A, Leonardi V et al. Treatment of metastatic breast cancer with vinorelbine and docetaxel. *Am J Clin Oncol*, 2006. 29(3):276-280.
28. Berruti A, Bitossi R, Gorzegno G et al. Paclitaxel, vinorelbine and 5-fluorouracil in breast cancer patients pretreated with adjuvant anthracyclines. Br J Cancer, 2005. 92(4):634-637.
29. Shamseddine A, Khalifeh M, Chehal A et al. A clinical phase II study of cisplatinum and vinorelbine (PVn) in advanced breast carcinoma (ABC). *Am J Clin Oncol*, 2005. 28(4):393-398.
30. Hortobagyi GN. Treatment of advanced breast cancer with gemcitabine and vinorelbine. *Oncology (Williston Park)*, 2001. 15(2S3):15-17.
31. Ejlertsen B, Mouridsen HT, Langkjer ST et al. Phase III study of intravenous vinorelbine in combination with epirubicin versus epirubicin alone in patients with advanced breast cancer: a Scandinavian Breast Group Trial (SBG9403). *J Clin Oncol*, 2004. 22(12):2313-2320.
32. Pegram MD, Konecny GE, O'Callaghan C et al, Rational combinations of trastuzumab with chemotherapeutic drugs used in the treatment of breast cancer. *J Nat Cancer Inst*, 2004. 96(10):739-749.
33. Jahanzeb M, Mortimer JE, Yunus F et al, Phase II trial of weekly vinorelbine and trastuzumab as first-line therapy in patients with HER2(+) metastatic breast cancer. *Oncologist*, 2002. 7(5):410-417.
34. Burstein HJ, Harris LN, Marcom PK et al, Trastuzumab and vinorelbine as first-line therapy for HER2-overexpressing metastatic breast cancer: multicenter phase II trial with clinical outcomes, analysis of serum tumor markers as predictive factors, and cardiac surveillance algorithm; *J Clin Oncol*, 2003. 21(15):2889-2895.
35. Chan A, Martin M, Untch M et al. Vinorelbine plus trastuzumab combination as first-line therapy for HER 2-positivve metastatic breast cancer patients: an international phase II trial. *Br J Cancer*, 2006. 95(7):788-793.
36. Papaldo P, Fabi A, Ferretti G et al. A phase II study on metastatic breast cancer patients treated with weekly vinorelbine with or without trastuzumab according to HER2 expression: changing the natural history of HER2-positive disease. *Ann Oncol*, 2006. 17(4):630-636.
37. Rahmani R, Bruno R, Iliadis A et al. Clinical pharmacokinetics of the antitumor drug navelbine (5'-noranhydrovinblastine). *Cancer Res*, 1987. 47(21):5796-5799.
38. Kobayashi S, Sakai T, Dalrymple PD et al. Disposition of the novel anti-cancer agent vinorelbine ditartrate following intravenous administration on mice, rats and dogs. *Arzneimittrl-Forschung*, 1993. 43:1367-1377.
39. Laveque D, Quoix E, Dumont P et al. Pulmonary distribution of vinorelbine in patients with non small cell lung cancer. *Cancer Chemother Pharmacol*, 1993. 33:176-178.
40. Beulz-Riche D, Grude P, Puozzo C et al. Characterization of human cytochrome P450 isoenzymes involved in the metabolism of vinorelbine. *Fundam Clin Pharmacol*, 2005. 19(5):545-553.
41. Marquet P, Lachetre G, Debord J et al. Pharmacokinetics of vinorelbine in man. *Eur J Clin Pharmacol*, 1992. 42:545-547.
42. Cvittovic E, Izzo J. The current and future place of Vinorelbine in cancer therapy. *Drugs*, 1992. 44(sup.4):36-45.
43. Navelbine Product Information.
44. Dubos C, Prevost JN, Brun J, Rousselot P. Infarctus myocardique et vinorelbine. *Rev Mal Resp*, 1991. 8:299-300.
45. Besenval M, Delgado M, Demarez JP, Krikorian A. Safety and tolerance of Navelbine in phase I-II clinical studies. *Semin Oncol*, 1989. 16(sup.4):37-40.
46. Wilhelm S, Chien DS: Bay 43-9006: Preclinical data. *Curr Pharm Des*, 2002. 8:2255-2257.
47. Beeram M, Patnaik A, Rowinsky EK. Raf: a strategic target for therapeutic development against cancer. *J Clin Oncol*, 2005. 23(27):6771-6790.
48. Dhanasekaran N, Premkumar RE. Signaling by dual specific kinases. *Oncogene*, 1998. 17:1447-1455.
49. Li S, Sedivy JM, Raf-1 protein kinase activates the NF-kappa B transcription factor by dissociating the cytoplasmic NF-kappa B-I kappa B complex. *Proc Natl Acad Sci U S A*, 1993. 90:9247-9251.
50. Wang HG, Miyashita T, Takayama S et al, Apoptosis regulation by interaction of Bcl-2 protein and Raf-1

kinase. *Oncogene*, 1994. 9:2751-2756.

51. Cornwell MM, Smith DE. A signal transduction pathway for activation of the mdr1 promoter involves the proto-oncogene c-raf kinase. *J Biol Chem*, 1993. 268:15347-15350.
52. Meamura M, Iino Y, Koibuchi Y et al. Mitogen-activated protein kinase cascade in breast cancer. *Oncology*, 1999. 57(sup.2):37-44.
53. Callans LS, Naama H, Khandelwal M et al. Raf-1 protein expression in human breast cancer cells. *Ann Surg Oncol*, 1995. 2(1):38-42.
54. Davies H, Bignell GR, Cox C et al. Mutations of the BRAF gene in human cancer. *Nature*, 2002. 417(6892):949-954.
55. Navolanic PM, Steelman LS, McCubrey JA. EGFR family signaling and its association with breast cancer development and resistance to chemotherapy (Review). *Int J Oncol*, 2003. 22(2):237-252.
56. Weinstein-Oppenheimer CR, Burrows C, Steelman LS, McCubrey JA. The effects of beta-estradiol on Raf activity, cell cycle progression and growth factor synthesis in the MCF-7 breast cancer cell line. *Cancer Biol Ther*, 2002.1(3):256-262.
57. Geiger T, Muller M, Monia BP, Fabbro D. Antitumor activity of a C-raf antisense oligonucleoide in combination with standard chemotherapeutic agents against various human tumors transplanted subcutaneously into nude mice. *Clin Cancer Res*, 1997. 3(7):1179-1185.
58. Leng Q, Mixson AJ. Small interfering RNA targeting Raf-1 inhibits tumor growth in vitro and in vivo. *Cancer Gene Ther*, 2005. 12(8):682-690.
59. Wilhelm SM, Carter C, Tang LY et al. BAY 43-9006 exhibits broad spectrum oral antitumor activity and targets the RAF/MEK/ERK pathway and receptor tyrosine kinases involved in tumor progression and angiogenesis. *Cancer Res*, 2004. 64:7099-7109.
60. Miller KD, Dul CL. Breast cancer: the role of angiogenesis and antiangiogenic therapy. *Hematol Oncol Clin North Am*, 2004. 18:1071-1086.
61. Jain RK, Duda DG, Clark JW, Loeffler JS. Lesson from phase III clinical trials on anti-VEGF therapy for cancer. *Nat Clin Pract Oncol*, 2006. 3:24-40.
62. Ferrara N. VEGF and the quest for tumour angiogenesis factors. *Nat Rev Cancer*, 2002. 795-803.
63. Waltenberger J, Claesson-Welsh L, Siegbahn, Shibuya M, Heldin CH. Different signal transduction properties of KDR and Flt1, two receptors for vascular endothelial growth factor. *J Biol Chem*, 1994. 269:26988-26995.
64. Millauer B, Longhi MP, Plate KH, et al. Dominant-negative inhibition of Flk-1 suppresses the growth of many tumor types in vivo. *Cancer Res*, 1996. 56:1615-1620.
65. Carter CA, Chen C, Brink C et al. Sorafenib is efficacious and tolerated in combination with cytotoxic or cytostatic agents in preclinical models of human non-small cell lung carcinoma. *Cancer Chemother Pharmacol*, 2007. 59(2):183-195.
66. Awada A, Hendlisz A, Gil T et al, Phase I safety and pharmacokinetics of BAY 43-9006 administered fro 21 days on/7 days off in patients with advanced, refractory solid tumours. *Br J Cancer*, 2005. 92:1855-1861.
67. Moore M, Hirte HW, Siu L et al, Phase I study to determine the safety and pharmacokinetics of the novel Raf kinase and VEGFR inhibitor BAY 43-9006, administered for 28 days on/7 days off in patients with advanced, refractory solid tumors. *Ann Oncol*, 2005. 16:1688-1694.
68. Clark JW, Eder JP, Ryan D et al, Safety and pharmacokinetics of the dual action Raf kinase and vascular endothelial growth factor receptor inhibitor, BAY 43-9006, in patients with advanced, refractory solid tumors. *Clin Cancer Res*, 2005. 11(15): 5472-5480.
69. Strumberg D, Richly H, Hilger RA et al, Phase I clinical and pharmacokinetic study of the novel Raf kinase and vascular endothelial growth factor receptor inhibitor BAY 43-9006 in patients with advanced refractory ssolid tumors. *J Clin Oncol*, 2005. 23(5):965-972.
70. Activity of the Raf kinase inhibitor BAY 43-9006 in patients with advanced solid tumors. *Clin Colorectal Cancer*, 2003. 3:16-18.
71. Russo P, Ratain MJ, Eisen T et al. Phase II placebo-controlled randomized discontinuation trial of sorafenib in patients with metastatic renal cell carcinoma. *Urol Oncol*, 2006. 24(6):560.
72. Loibi S, Bianchi G, Zamagni C et al. Sorafenib (BAY 43-9006) in patients with metastatic breast cancer – a Phase II multicentre open trial. Proc 27 Deutscher Krebskongress, 2006. PO032.
73. Lyons JF, Wilhelm S, Hibner B et al. Discovery of a novel Raf kinase inhibitor. *Endocr Relat Cancer*, 2001. 8:219-225.

74. Strumberg D, Voliotis D, Moeller JG et al. Results of phase I pharmacokinetic and pharmacodynamic studies of the Raf kinase inhibitor BAY 43-9006 in patients with solid tumors. *Int J Clin Pharmacol Ther*, 2002. 40:580-581.

75. Hilger RA, Kredke S, Hedley D et al. Inhibition of ERK phosphorilation in patients treated with the Raf kinase inhibitor BAY 43-9006. *Eur J Cancer*, 2002. 38:S52-S53.

76. Strumberg D, Awada A, Hirte h et al. Pooled safety analysis of BAY 43-9006 (sorafenib) monotherapy in patients with advanced solid tumors: is rash associated with treatment outcome? *Eur J Cancer*, 2006. 42(4):548-556.

77. Ahmad T, Eisen T. Kinase inhibition with BAY 43-9006 in renal cell carcinoma. *Clin Cancer Res*, 2004; 10:S6388-S6392.

78. Flaherty KT, Brose M, Schuchter L et al. Phase I/II trial of BAY-43-9006, carboplatin (C) and pacliatxel (P) demonstrates preliminary antitumor activity in the expansion cohort of patients with metastatic melanoma. *Proc ASCO*, 2004, abstr 7507.

79. Kupsch P, Henning BF, Passarge K et al. Results of a phase I trial of sorafenib (BAY 43-9006) in combination with oxaliplatin in patients with refractory solid tumors, including colorectal cancer. *Clin Colorectal Cancer*, 2005. 5(3):188-196.

80. Siu LL, Awada A, Takimoto CH et al. Phase I trial os Sorafenib and Gemcitabine in advanced solid tumors with an expanded cohort in advanced pancreatic cancer. *Clin Cancer Res*, 2006. 12(1):144-151.

81. Davis JM, Navolanic PM, Weinstein-Oppenheimer CR et al. Raf-1 and Bcl-2 induce distinct and common pathways that contribute to breast cancer drug resistance. *Clin Cancer Res*, 2003. 9(3):1161-1170.

82. Mewani RR, Tang W, Rahman A et al. Enhanced therapeutic effects of doxorubicin and paclitaxel in combination with liposome-entrapped ends-modified raf antisense oligonucleotide against human prostate, lung and breast tumor models. *Int J Oncol*, 2004. 24(5):1181-1188.

83. Vincent P, Zhang X, Chen M et al, Preclinical chemotherapy with the raf kinase inhibitor BAY 43-9006 in combination with gefitinib, vinorelbine, gemcitabine and doxorubicin. Presented at Am Assoc Cancer Res Annual Meeting, 2003.

84. Jain RK. Normalizing tumor vasculature with anti-angiogenic therapy: a new paradigm for combination therapy. *Nat Med*, 2001. 7:987–9.

85. Wildiers H, Guetens G, De Boeck G, et al. Effect of antivascular endothelial growth factor treatment on the intratumoral uptake of CPT-11. *Br J Cancer*, 2003. 88:1979–86.

86. Klement G, Huang P, Mayer B et al: Differences in therapeutic indexes of combination metronomic chemotherapy and an anti-VEGFR-2 antibody in multidrug-resistant human breast cancer xenografts. *Clin Cancer Res*, 2002. 8:221-232.

87. Burstein HJ, Keshaviah A, Baron A et al. Trastuzumab and vinorelbine or taxane chemotherapy for HER2+ metastatic breast cancer: The TRAVIOTA study. *Proc ASCO*, 2006. Abstr 650.

88. Joensuu H, Kellokumpu-Lehtinen PL, Bono P et al. Adjuvant docetaxel or vinorelbine with or without trastuzumab for breast cancer. *N Engl J Med*, 2006. 354:809-820.

PART III: APPENDIXES

APPENDIX A

DECLARATION OF HELSINKI
World Medical Association Declaration of Helsinki

Recommendations guiding physicians in biomedical research involving human subjects. Adopted by the 18th World Medical Assembly, Helsinki, Finland, June 1964, and amended by the 29th World Medical Assembly, Tokyo, Japan, October 1975, 35th World Medical Assembly, Venice, Italy, October 1983, and the 41st World Medical Assembly, Hong Kong, September 1989 and the 48th General Assembly, Somerset West, Republic of South Africa, October 1996.

INTRODUCTION

It is the mission of the physician to safeguard the health of the people. His or her knowledge and conscience are dedicated to the fulfillment of this mission.

The Declaration of Geneva of the World Medical Association binds the physician with the words, "The health of my patient will be my first consideration", and the International Code of Medical Ethics declares that "A physician shall act only in the patient's interest when providing medical care which may have the effect of weakening the physical and mental condition of the patient."

The purpose of biomedical research involving human subjects must be to improve diagnostic, therapeutic or prophylactic procedures and the understanding of the ætiology and pathogenesis of disease.

In current medical practice most diagnostic, therapeutic or prophylactic procedures involve hazards. This applies especially to biomedical research.

Medical progress is based on research which ultimately must rest in part on experimentation involving human subjects.

In a field of biomedical research a fundamental distinction must be recognized between medical research in which the aim is essentially diagnostic or therapeutic for a patient and medical research, the essential object of which is purely scientific and without implying direct diagnostic or therapeutic value to the person subjected to the research.

Special caution must be exercised in the conduct of research, which may affect the environment, and the welfare of animals used for research must be respected.

Because it is essential that the results of laboratory experiments be applied to human beings to further scientific knowledge and to help suffering humanity, the World Medical

Association has prepared the following recommendations as a guide to every physician in biomedical research involving human subjects. They should be kept under review in the future. It must be stressed that the standards as drafted are only a guide to physicians all over the world. Physicians are not relieved from criminal, civil and ethical responsibilities under the laws of their own countries.

BASIC PRINCIPLES

1. Biomedical research involving human subjects must conform to generally accepted scientific principles and should be based on adequately performed laboratory and animal experimentation and on a thorough knowledge of the scientific literature.
2. The design and performance of each experimental procedure involving human subjects should be clearly formulated in an experimental protocol which should be transmitted for consideration, comment, and guidance to a specially appointed committee independent of the investigator and the Sponsor provided that this independent committee is in conformity with the laws and regulations of the country in which the research experiment is performed.
3. Biomedical research involving human subjects should be conducted only by scientifically qualified persons and under the supervision of a clinically competent medical person. The responsibility for the human subject must always rest with the medically qualified person and never rest on the subject of the research, even though the subject has given his or her consent.
4. Biomedical research involving human subjects cannot legitimately be carried out unless the importance of the objective is in proportion to the inherent risk to the subject.
5. Every biomedical research project involving human subjects should be preceded by careful assessment of predictable risks in comparison with the foreseeable benefits to the subject or to others. Concern for the interest of the subject must always prevail over the interest of science and society.
6. The right of the research subject to safeguard his or her integrity must always be respected. Every precaution should be taken to respect the privacy of the subject and to minimize the impact of the study on the subject's physical and mental integrity and on the personality of the subject.
7. Physicians should abstain from engaging in research projects involving human subjects unless they are satisfied that the hazards involved are believed to be predictable. Physicians should cease any investigation if the hazards are found to outweigh the potential benefits.
8. In publication of the results of his or her research, the physician is obliged to preserve the accuracy of the results. Reports on experimentation not in accordance with the principles laid down in this Declaration should not be accepted for publication.
9. In any research on human beings, each potential subject must be adequately informed of the aims, methods, anticipated benefits, and potential hazards of the study and the discomfort it may entail. or she should be informed that he or she is at liberty to abstain from participation in the study and that he or she if free to withdraw his or her consent to participation at any time. The physician should

then obtain the subject's freely-given informed consent, preferably in writing.

10. When obtaining informed consent for the research project the physician should be particularly cautious if the subject is in a dependent relationship to him or her or may consent under duress. In that case the informed consent should be obtained by a physician who is not engaged in the investigation and who is completely independent of this official relationship.
11. In the case of legal incompetence, informed consent should be obtained from the legal guardian in accordance with national legislation. Where physical or mental incapacity makes it impossible to obtain informed consent, or when the subject is a minor, permission from the responsible relative replaces that of the subject in accordance with national legislation. Whenever the minor child is in fact able to give a consent, the minor'sconsent must be obtained in addition to the consent of the minor's legal guardian.
12. The research protocol should always contain a statement of the ethical consideration involved and should indicate that the principles enunciated in the present Declaration are complied with.

Medical Research Combined With Professional Care (Clinical Research)

1. In the treatment of the sick person, the physician must be free to use a new diagnostic and therapeutic measure, if in his or her judgment it offers hope of saving life, reestablishing health or alleviating suffering.
2. The potential benefits, hazards, and discomfort of a new method should be weighed against the advantages of the best current diagnostic and therapeutic methods.
3. In any medical study, every patient – including those of a control group, if any – should be assured of the best proven diagnostic and therapeutic method. This does not exclude the use of inert placebo in studies where no proven diagnostic or therapeutic method exists.
4. The refusal of the patient to participate in a study must never interfere with the physician-patient relationship.
5. If a physician considers it essential not to obtain informed consent the specific reasons for this proposal should be stated in the experimental protocol for transmission to the independent committee (1.2).
6. The physician can combine medical research with professional care, the objective being the acquisition of new medical knowledge, only to the extent that medical research is justified by its potential diagnostic or therapeutic value for the patient.

Non-Therapeutic Biomedical Research Involving Human Subjects (Non-Clinical Biomedical Research)

In the purely scientific application of medical research carried out on a human being, it is the duty of the physician to remain the protector of life and health of that person on whom biomedical research is being carried out.

The subjects should be volunteers – either healthy persons or patients for whom the

experimental design is not related to the patient's illness.

The investigator of the investigating team should discontinue the research if in his/her or their judgment it may, if continued, be harmful to the individual.

In research on man, the interest of science and society should never take precedence over considerations related to the well-being of the subject.

APPENDIX B

ECOG PERFORMANCE STATUS

Grade Performance Status as follow:

0: Able to carry out all normal activity without restriction.

1: Restricted in physically strenuous activity but ambulatory and able to do light work.

2: Ambulatory and capable of all self-care but unable to carry out any work. Up and about more than 50% of waking hours.

3: Capable of only limited self-care, confined to bed or chair more than 50% of waking hours.

4: Completely disabled. Cannot carry on any self-care. Totally confined to bed or chair.

APPENDIX C

CREATININE CLEARANCE

Normal Creatinine Clearance for Women:

Average: **95 ml/min** / 1.73 m^2 (+/-20) [or 135 Liters/day

Range: 88-128 ml/min/ 1.73 m^2

If plasma creatinine is relatively stable, creatinine clearance can be estimated through Cockcroft-Gault formula for GFR estimate:

Estimated Creatinine Clearance (ml/min) =

[140 – age (yr)] x weight (kg) / [72 x serum creatinine (mg/dL)] x 0.85 *

* *(multiply by 0.85 for women)*

In case of:

1. Pregnancy;
2. Severely underweight or overweight;
3. Very young and very old;
4. Malnutrition;
5. Skeletal muscle disorders, paraplegia, quadriplegia

Calculation of 24 hour Creatinine Clearance is indicated, as follows:

Calculated Creatinine Clearance (ml/min) =

(uCr x uV) / (sCr x 1440)

where:

- uCr is Urine Creatinine in mg/dl;
- sCr is Serum Creatinine in mg/dl;
- uV is 24 hour urine volume in ml;
- 1440 represents number of minutes in 24 hours.

APPENDIX D

The common toxicity of the NCI CTCAE (Version 3.0, dated 12 December 2003) may be reviewed on-line at

http://ctep.cancer.gov/reporting/ctc.html

Click on link to CTCAE.

APPENDIX E

Response Evaluation Criteria in Solid Tumors (RECIST) Quick Reference:

Eligibility

Only patients with measurable disease at baseline should be included in protocols where objective tumor response is the primary endpoint.

Measurable disease: the presence of at least one measurable lesion. If the measurable disease is restricted to a solitary lesion, its neoplastic nature should be confirmed by cytology/histology.

Measurable lesions: lesions that can be accurately measured in at least one dimension with longest diameter □20 mm using conventional techniques or □10 mm with spiral CT scan.

Non-measurable lesions: all other lesions, including small lesions (longest diameter <20 mm with conventional techniques or <10 mm with spiral CT scan), i.e., bone lesions, leptomeningeal disease, ascites, pleural/pericardial effusion, inflammatory breast disease, lymphangitis cutis/pulmonis, cystic lesions, and also abdominal masses that are not confirmed and followed by imaging techniques.

All measurements should be taken and recorded in metric notation, using a ruler or calipers. All baseline evaluations should be performed as closely as possible to the beginning of treatment and never more than 4 weeks before the beginning of the treatment.

The same method of assessment and the same technique should be used to characterize each identified and reported lesion at baseline and during follow-up.

Clinical lesions will only be considered measurable when they are superficial (e.g., skin nodules and palpable lymph nodes). For the case of skin lesions, documentation by color photography, including a ruler to estimate the size of the lesion, is recommended.

Methods of Measurement

CT and MRI are the best currently available and reproducible methods to measure target lesions selected for response assessment. Conventional CT and MRI should be performed with cuts of 10 mm or less in slice thickness contiguously. Spiral CT should be performed using a 5 mm contiguous reconstruction algorithm. This applies to tumors of the chest, abdomen and pelvis. Head and neck tumors and those of extremities usually require specific protocols.

Lesions on chest X-ray are acceptable as measurable lesions when they are clearly defined and surrounded by aerated lung. However, CT is preferable.

When the primary endpoint of the study is objective response evaluation, ultrasound (US) should not be used to measure tumor lesions. It is, however, a possible alternative to clinical measurements of superficial palpable lymph nodes, subcutaneous lesions and thyroid nodules. US might also be useful to confirm the complete disappearance of superficial lesions usually assessed by clinical examination.

The utilization of endoscopy and laparoscopy for objective tumor evaluation has not yet been fully and widely validated. Their uses in this specific context require sophisticated equipment and a high level of expertise that may only be available in some centers. Therefore, the utilization of such techniques for objective tumor response should be restricted to validation purposes in specialized centers. However, such techniques can be useful in confirming complete pathological response when biopsies are obtained.

Tumor markers alone cannot be used to assess response. If markers are initially above the upper normal limit, they must normalize for a patient to be considered in complete clinical response when all lesions have disappeared.

Cytology and histology can be used to differentiate between PR and CR in rare cases (e.g., after treatment to differentiate between residual benign lesions and residual malignant lesions in tumor types such as germ cell tumors).

Baseline documentation of "Target" and "Non-Target" lesions

All measurable lesions up to a maximum of five lesions per organ and 10 lesions in total, representative of all involved organs should be identified as *target lesions* and recorded and measured at baseline.

Target lesions should be selected on the basis of their size (lesions with the longest diameter) and their suitability for accurate repeated measurements (either by imaging techniques or clinically).

A sum of the longest diameter (LD) for *all target lesions* will be calculated and reported as the baseline sum LD. The baseline sum LD will be used as reference by which to characterize the objective tumor.

All other lesions (or sites of disease) should be identified as *non-target lesions* and should also be recorded at baseline. Measurements of these lesions are not required, but the presence or absence of each should be noted throughout follow-up.

Response Criteria

Evaluation of target lesions

* Complete Response (CR):
Disappearance of all target lesions.

* Partial Response (PR):
At least a 30% decrease in the sum of the LD of target lesions, taking as reference the baseline sum LD.

* Progressive Disease (PD):
At least a 20% increase in the sum of the LD of target lesions, taking as reference the smallest sum LD recorded since the treatment started or the appearance of one or more new lesions.

* Stable Disease (SD):
Neither sufficient shrinkage to qualify for PR, nor sufficient increase to qualify for PD, taking as reference the smallest sum LD since the treatment started.

Evaluation of non-target lesions

* Complete Response (CR):
Disappearance of all non-target lesions and normalization of tumor marker level

* Incomplete Response/Stable Disease (SD):
Persistence of one or more non-target lesion(s) or/and maintenance of tumor marker level above the normal limits

* Progressive Disease (PD):

Appearance of one or more new lesions and/or unequivocal progression of existing non-target lesions. Although a clear progression of "non target" lesions only is exceptional, in such circumstances, the opinion of the treating physician should prevail and the progression status should be confirmed later on by the review panel (or study chair).

Evaluation of best overall response

The best overall response is the best response recorded from the start of the treatment until disease progression/recurrence (taking as reference for PD the smallest measurements recorded since the treatment started). In general, the patient's best response assignment will depend on the achievement of both measurement and confirmation criteria.

Target lesions	Non-Target lesions	New Lesions	Overall response
CR	CR	No	**CR**
CR	Incomplete response/SD	No	**PR**
PR	Non-PD	No	**PR**
SD	Non-PD	No	**SD**
PD	Any	Yes or No	**PD**
Any	PD	Yes or No	**PD**
Any	Any	Yes	**PD**

Patients with a global deterioration of health status requiring discontinuation of treatment without objective evidence of disease progression at that time should be classified as having "symptomatic deterioration". Every effort should be made to document the objective progression even after discontinuation of treatment.

In some circumstances it may be difficult to distinguish residual disease from normal tissue. When the evaluation of complete response depends on this determination, it is recommended that the residual lesion be investigated (fine needle aspirate/biopsy) to confirm the complete response status.

Confirmation

The main goal of confirmation of objective response is to avoid overestimating the response rate observed. In cases where confirmation of response is not feasible, it should be made clear when reporting the outcome of such studies that the responses are not confirmed.

To be assigned a status of PR or CR, changes in tumor measurements must be confirmed by repeat assessments that should be performed no less than 4 weeks after the criteria for response are first met. Longer intervals as determined by the study protocol may also be appropriate.

In the case of SD, follow-up measurements must have met the SD criteria at least once after study entry at a minimum interval (in general, not less than 6-8 weeks) that is defined in the study protocol

Duration of overall response

The duration of overall response is measured from the time measurement criteria are met for CR or PR (whichever status is recorded first) until the first date that recurrence or PD is objectively documented, taking as reference for PD the smallest measurements recorded since the treatment started.

Duration of stable disease

SD is measured from the start of the treatment until the criteria for disease progression are met, taking as reference the smallest measurements recorded since the treatment started.
The clinical relevance of the duration of SD varies for different tumor types and grades. Therefore, it is highly recommended that the protocol specify the minimal time interval required between two measurements for determination of SD. This time interval should take into account the expected clinical benefit that such a status may bring to the population under study.

Response review

For trials where the response rate is the primary endpoint it is strongly recommended that all responses be reviewed by an expert(s) independent of the study at the study's completion. Simultaneous review of the patients' files and radiological images is the best approach.

Reporting of results

All patients included in the study must be assessed for response to treatment, even if there are major protocol treatment deviations or if they are ineligible. Each patient will be assigned one of the following categories: 1) complete response, 2) partial response, 3) stable disease, 4) progressive disease, 5) early death from malignant disease, 6) early

death from toxicity, 7) early death because of other cause, or 9) unknown (not assessable, insufficient data).

All of the patients who met the eligibility criteria should be included in the main analysis of the response rate. Patients in response categories 4-9 should be considered as failing to respond to treatment (disease progression). Thus, an incorrect treatment schedule or drug administration does not result in exclusion from the analysis of the response rate. Precise definitions for categories 4-9 will be protocol specific.
All conclusions should be based on all eligible patients.

Sub-analyses may then be performed on the basis of a subset of patients, excluding those for whom major protocol deviations have been identified (e.g., early death due to other reasons, early discontinuation of treatment, major protocol violations, etc.). However, these sub-analyses may not serve as the basis for drawing conclusions concerning treatment efficacy, and the reasons for excluding patients from the analysis should be clearly reported.

The 95% confidence intervals should be provided.

APPENDIX F

INSTRUCTIONS FOR HANDLING OF PHARMACOKINETIC SAMPLES FOR VINORELBINE, SORAFENIB AND METABOLITE M2

Venous blood samples (7 mL each) will be collected by venipuncture or through a heparin lock into tubes containing lithium heparin.

Plasma sample processing:

1. Within 30 minutes after sampling, the blood will be centrifuged.
2. Each sample will be **centrifuged for 10 minutes at 1500g** to separate the plasma.
3. A minimum of 2 mL of plasma will be transferred to 5 mL screw cap polypropylene test tubes (preferably Nalgene Cryovial, Cat. No. 5000-0050, with external thread, volume indicator, and freestanding flat bottom).
4. Test tubes must be labeled with the study number, the compound name, the subject's random number, and the date and time of sampling relative to dosing.

5. The **test tube labels must be able to adhere to the polypropylene tube surface at the ultra low temperature of (< -80° C)** achieved during shipment on dry ice; it is advisable to secure the label to the tube with transparent freezer tape.

6. The test tubes of plasma must be **frozen in an upright position and kept frozen at approximately -20° C** until ready for shipment to the sponsor.

Plasma Sample Shipment:

- **Samples should be sorted by subject,** so that all samples from a given subject are readily accessible.
- Each set of samples should be wrapped with paper towels (or equivalent), taped **and packed in a separate container or freezer bag**. The freezer bag should also be taped to prevent movement of the tubes within the bag.
- An **inventory of samples must be included (PK Log templates)** as an indication of what is included in the shipment (a copy of the template provided would be sufficient).
- Packaging and shipment of samples must be in accordance with the guidelines set forth by the International Air Transport Association (IATA).
- The frozen samples will be shipped to Bayer by **overnight delivery** (specifically, Federal Express), packed in **sufficient dry ice (at least 10 lbs) to ensure that they remain frozen for at least two days.**
- Shipment should be **scheduled for early in the week** (Mondays, Tuesdays, or Wednesdays) to ensure that the samples do not arrive on a weekend.
- Vipin Agarwal, PhD must be **notified by FAX at (203) 407-0703** or email (vipin.agarwal.b@bayer.com) at least one day in advance of shipping the samples.
- All frozen samples from Study are to be shipped to:

Vipin Agarwal, PhD
NorthEast Bioanalytical Laboratories
925 Sherman Avenue
Hamden, CT 06514
Tel: 203-407-0400
Fax: 203-407-0703
